# Comprehensive cross-disorder analyses of *CNTNAP2* suggest it is unlikely to be a primary risk gene for psychiatric disorders

**DOI:** 10.1371/journal.pgen.1007535

**Published:** 2018-12-26

**Authors:** Claudio Toma, Kerrie D. Pierce, Alex D. Shaw, Anna Heath, Philip B. Mitchell, Peter R. Schofield, Janice M. Fullerton

**Affiliations:** 1 Neuroscience Research Australia, Sydney, Australia; 2 School of Medical Sciences, University of New South Wales, Sydney, Australia; 3 School of Psychiatry, University of New South Wales, Sydney, Australia; 4 Black Dog Institute, Prince of Wales Hospital, Sydney, Australia; Stanford University School of Medicine, UNITED STATES

## Abstract

The contactin-associated protein-like 2 *(CNTNAP2)* gene is a member of the neurexin superfamily. *CNTNAP2* was first implicated in the cortical dysplasia-focal epilepsy (CDFE) syndrome, a recessive disease characterized by intellectual disability, epilepsy, language impairments and autistic features. Associated SNPs and heterozygous deletions in *CNTNAP2* were subsequently reported in autism, schizophrenia and other psychiatric or neurological disorders. We aimed to comprehensively examine evidence for the role of *CNTNAP2* in susceptibility to psychiatric disorders, by the analysis of multiple classes of genetic variation in large genomic datasets. In this study we used: i) summary statistics from the Psychiatric Genomics Consortium (PGC) GWAS for seven psychiatric disorders; ii) examined all reported *CNTNAP2* structural variants in patients and controls; iii) performed cross-disorder analysis of functional or previously associated SNPs; and iv) conducted burden tests for pathogenic rare variants using sequencing data (4,483 ASD and 6,135 schizophrenia cases, and 13,042 controls). The distribution of CNVs across *CNTNAP2* in psychiatric cases from previous reports was no different from controls of the database of genomic variants. Gene-based association testing did not implicate common variants in autism, schizophrenia or other psychiatric phenotypes. The association of proposed functional SNPs rs7794745 and rs2710102, reported to influence brain connectivity, was not replicated; nor did predicted functional SNPs yield significant results in meta-analysis across psychiatric disorders at either SNP-level or gene-level. Disrupting *CNTNAP2* rare variant burden was not higher in autism or schizophrenia compared to controls. Finally, in a CNV mircroarray study of an extended bipolar disorder family with 5 affected relatives we previously identified a 131kb deletion in *CNTNAP2* intron 1, removing a FOXP2 transcription factor binding site. Quantitative-PCR validation and segregation analysis of this CNV revealed imperfect segregation with BD.

This large comprehensive study indicates that *CNTNAP2* may not be a robust risk gene for psychiatric phenotypes.

## Author summary

Genetic mutations that disrupt both copies of the *CNTNAP2* gene lead to severe disease, characterized by profound intellectual disability, epilepsy, language difficulties and autistic traits, leading to the hypothesis that this gene may also be involved in autism given some overlapping clinical features with this disease. Indeed, several large DNA deletions affecting one of the two copies of *CNTNAP2* were found in some patients with autism, and later also in patients with schizophrenia, bipolar disorder, ADHD and epilepsy, suggesting that this gene was implicated in several psychiatric or neurologic diseases. Other studies considered genetic sequence variations that are common in the general population, and suggested that two such sequence variations in *CNTNAP2* predispose to psychiatric diseases by influencing the functionality and connectivity of the brain. To better understand the involvement of *CNTNAP2* in risk of mental illness, we performed several genetic analyses using a series of large publicly available or in-house datasets, comprising many thousands of patients and controls. Furthermore, we report the deletion of one copy of *CNTNAP2* in two patients with bipolar disorder and one unaffected relative from an extended family where five relatives were affected with this condition. Despite the previous consideration of *CNTNAP2* as a strong candidate gene for autism or schizophrenia, we show little evidence across multiple classes of DNA variation, that *CNTNAP2* is likely to play a major role in risk of psychiatric diseases.

## Introduction

The contactin-associated protein-like 2 (*CNTNAP2*) is located on chromosome 7q35-36.1, and consists of 24 exons spanning 2.3Mb, making it one of the largest protein coding genes in the human genome. This gene encodes the CASPR2 protein, related to the neurexin superfamily, which localises with potassium channels at the juxtaparanodal regions of the Ravier nodes in myelinated axons, playing a crucial role in the clustering of potassium channels required for conduction of axon potentials [1]. *CNTNAP2* is expressed in the spinal cord, prefrontal and frontal cortex, striatum, thalamus and amygdala; this pattern of expression is preserved throughout the development and adulthood [2, 3]. Its function is related to neuronal migration, dendritic arborisation and synaptic transmission [4]. The crucial role of *CNTNAP2* in the human brain became clear in 2006 when Strauss *et al*, reported homozygous mutations in Old Order Amish families segregating with a severe Mendelian condition, described as cortical dysplasia-focal epilepsy (CDFE) syndrome (OMIM 610042) [5]. In 2009, additional patients with recessive mutations in *CNTNAP2* were reported, with clinical features resembling Pitt-Hopkins syndrome [6]. To date 33 patients, mostly from consanguineous families, have been reported with homozygous or compound deletions and truncating mutations in *CNTNAP2* [5–9], and are collectively described as having CASPR2 deficiency disorder [7]. The common clinical features in this phenotype include severe intellectual disability (ID), seizures with age of onset at two years and concomitant speech impairments or language regression. The phenotype is often accompanied by dysmorphic features, autistic traits, psychomotor delay and focal cortical dysplasia.

*CNTNAP2* is also thought to contribute to diverse phenotypes in patients with interstitial or terminal deletions at 7q35 and 7q36. Interstitial or terminal deletions encompassing *CNTNAP2* and several other genes have been described in individuals with ID, seizures, craniofacial anomalies, including microcephaly, short stature and absence of language [10]. The severe language impairments observed in patients with homozygous mutations or karyotypic abnormalities involving *CNTNAP2* suggested a possible functional interaction with *FOXP2*, a gene for which heterozygous mutations lead to a monogenic form of language disorder [11]. Interestingly, *Vernes et al*., found that the FOXP2 transcription factor has a binding site in intron 1 of *CNTNAP2*, regulating its expression [12]. Considering that a large proportion of autistic patients show language impairments and most individuals with homozygous mutations in *CNTNAP2* manifest autistic features, several studies investigated the potential involvement of *CNTNAP2* in autism spectrum disorder (ASD). In particular, two pioneering studies showed that single nucleotide polymorphism (SNP) markers rs2710102 and rs7794745 were associated with risk of ASD [13, 14]. These studies were the first implicating *CNTNAP2* in autism, and opened a chapter of additional analyses in ASD and other psychiatric phenotypes during the next decade. In subsequent studies, rs2710102 was implicated in early language acquisition in the general population [15], and showed functional effects on brain activation in neuroimaging studies [16–19]. Furthermore, genotypes at rs7794745 were associated with reduced grey matter volume in the left superior occipital gyrus in two independent studies [20, 21], and alleles of this SNP were reported to affect voice-specific brain function [22]. Genetic associations with ASD for these, and several other SNPs in *CNTNAP2*, have been reported in a number of studies [23–28]. Along with the first reports of SNPs associated with ASD, copy number variant (CNV) deletions have also been described in ID or ASD patients, which were proposed to be highly penetrant disease-causative mutations [13, 29–38]. To better understand the role of *CNTNAP2* in ASD pathophysiology, knockout mice were generated. Studies of these mice reported several neuronal defects when both copies of *CNTNAP2* are mutated: abnormal neuronal migration, reduction of GABAergic interneurons, deficiency in excitatory neurotransmission, and the delay of myelination in the neocortex [2, 39, 40].

These intriguing findings prompted additional investigations of *CNTNAP2* across other psychiatric disorders or language-related traits, with additional reports of SNPs being associated with schizophrenia (SCZ), bipolar disorder (BD), specific language impairment (SLI) and several other phenotypes or traits [12, 15, 41–50]. Consequently, other studies reported CNV deletions in *CNTNAP2* in schizophrenia [51, 52], bipolar disorder [52–54], and ADHD [55]; but also in neurological disorders, especially epilepsy [56–61], and language-related phenotypes [62–65]. Interestingly, several of these structural variants were found in intron 1 of *CNTNAP2*, encompassing the FOXP2 transcription factor binding site. Epilepsy is clinically frequent in psychiatric disorders, especially schizophrenia and bipolar disorder [66–69], and is present in approximately 20% of autistic patients [70, 71]. Similarly, cognitive deficits involving language-related domains are also comorbid traits in schizophrenia and bipolar disorder [67, 72–74], and are common clinical features in ASD [75], with many ASD patients remaining non-verbal throughout life [75, 76].

While *CNTNAP2* is now considered a strong candidate gene for ASD and psychiatric disease more generally (summarised in Table 1), several of these early supportive studies were performed with limited sample sizes, or were individual case reports which lacked comparison with control individuals, hence providing circumstantial evidence as a psychiatric risk gene. We therefore aimed in this current study to perform systematic genetic analyses with large datasets to examine the evidence for a role of the *CNTNAP2* gene in multiple psychiatric phenotypes–performing a comprehensive analysis of common and rare variants, CNVs and *de novo* mutations–using both publicly available datasets and in-house data.

**Table 1:** Overview of studies implicating common alleles, structural variants or rare variants of *CNTNAP2* in psychiatric disorders.

Psychiatric phenotype	Variant described	Reference
Autism Spectrum Disorder	Associated SNP	Alarcon et al., 2008 [13]; Arking et al., 2008 [14]; Steer et al., 2010 [23]; Anney et al., 2012 [27]; Sampath et al., 2013 [26]; Poot, 2014 [28]; Chiocchetti et al., 2015 [25]; Nascimento et al., 2016 [24].
	CNV deletion	Alarcon et al., 2008 [13]; Poot et al., 2010 [34]; Nord et al., 2011 [37]; Girirajan et al., 2013 [35]; Egger et al., 2014 [38].
	CNV duplication	Prasad et al., 2012 [36]; Girirajan et al., 2013 [35].
	Rearrangement (Inversion)	Bakkaloglu et al., 2008 [33].
	Rare variants	Bakkaloglu et al., 2008 [33]; Chiocchetti et al., 2015 [25].
Schizophrenia	Associated SNP	Wang et al., 2010 [41]; Ji et al., 2013 [49]; Chen et al. 2015, [45];
	CNV deletion	Malhotra et al., 2011 [52]; Friedman et al., 2008 [51].
Bipolar Disorder	Associated SNP	Wang et al., 2010 [41].
	CNV deletion	Zhang et al., 2009 [53]; Malhotra et al., 2011 [52]; Lee et al., 2015 [54].
	CNV duplication	Malhotra et al., [52].
Attention Deficit Hyperactivity Disorder	CNV duplication	Elia et al., 2010 [55].
Major Depressive Disorder	Associated SNP	Ji et al., 2013 [49]; Wray et al., 2012 [44].
Alcohol dependence	Associated SNP	Zhong et al., 2005 [42].

## Results

### Analysis of *CNTNAP2* common single nucleotide variation in the susceptibility of psychiatric disorders

During the last decade, several association studies have been performed to assess the role of common variants of *CNTNAP2* in autism or speech-related phenotypes [12–15, 23–28, 46–48, 50], as well as several other psychiatric phenotypes [41–45, 49]. In Table 2, we summarise all markers found significantly associated in these previous studies, and report the corresponding *P-value* from the Psychiatric Genomics Consortium GWAS for seven major psychiatric disorders: ADHD, anorexia nervosa, ASD, bipolar disorder, MDD, OCD and schizophrenia. Nominal associations were found with ASD for the following markers: rs802524 (*P* = 0.016), rs802568 (*P* = 0.008), rs17170073 (*P* = 0.008), and rs2710102 (*P* = 0.036; which is highly correlated with 4 SNPs: rs759178, rs1922892, rs2538991, rs2538976). Furthermore, nominal association was also observed with schizophrenia for rs1859547 (*P* = 0.044); with ADHD for rs1718101 (*P* = 0.038); with MDD for rs12670868 (*P* = 0.047), rs17236239 (*P* = 0.006), rs4431523 (*P* = 0.001); and with anorexia nervosa for rs700273 (*P* = 0.013). The nominal association with ASD at rs1770073 and rs2710102 represents the only case in which the association in the original report replicates in the PGC dataset for the same phenotype. The two SNPs rs7794745 and rs2710102, which were repeatedly reported as being associated in earlier studies with smaller sample size and proposed to be functional SNPs, were not strongly associated with any phenotype (the most significant signal being *P* = 0.036 for rs2710102 in ASD). None of those associations survived corrections for multiple comparisons (Table 2).

**Table 2:** Common SNPs in *CNTNAP2* previously reported to be associated in psychiatric diseases, and their evidence for association in PGC datasets.

			PGC Association Results (*P-Value*)						
SNP	Location	Disease (Ref)	ASD	SCZ	BD	ADHD	MDD	AN	OCD
rs34712024	Promoter	ASD [25]	0.672^	0.45	0.099	0.442	N/A	0.283	0.295
rs802524	Intron 1	SCZ, BD [41]	**0.016**^	0.081	0.058	0.210	0.070	0.143	0.039
rs802568	Intron 1	SCZ, BD [41]	**0.008**^	0.061	0.312	0.047	0.054	0.321	0.279
rs17170073	Intron 1	ASD [26]	**0.008**	0.903	0.558	0.883	0.306	0.031	0.101
rs1718101	Intron 1	ASD [27]	0.076^	0.257	0.215	**0.038**	0.255	0.243	0.029
rs700273	Intron 1	ALD [42]	0.840	0.655	0.837	0.544	0.338	**0.013**	0.554
*rs7794745*	Intron 2	ASD [14, 23, 24]	0.906	0.734	0.498	0.393	0.173	0.877	0.503
rs10251794	Intron 3	OPN [43]	0.301	0.365	0.155	0.452	**0.047** (rs12670868)	0.648	0.351
rs7804520	Intron 3	ASD [28]	0.378	0.277	0.236	0.155	0.568	0.506	0.682
rs1603450	Intron 8	LAN [15]	0.445	0.166	0.643	0.141	0.010	0.951	0.577
rs826824	Intron 9	MDD (male only) [44]	0.218	0.181	0.256	0.317	0.266	0.736	0.199
rs1859547	Intron 11	SCZ [45]	0.697	**0.044**	0.431	0.225	0.939	0.729	0.154
rs851715^**&**^	Intron 13	SLI [12]	0.448	0.496	0.572	0.067	0.601	0.920	0.411
rs10246256^**&**^	Intron 13	SLI [12, 46]	0.429	0.613	0.508	0.070	0.601 (rs851715)	0.871	0.454
*rs2710102*^***#***^	Intron 13	ASD, SLI, DYS, SM, ANX, LAN, MDD [12, 13, 15, 23, 46–49]	**0.036**	0.893	0.801	0.911	0.346	0.383	0.351
rs759178^***#***^	Intron 13	SLI, LAN [12, 15]	**0.037**	0.890	0.799	0.929	0.332	0.363	0.347
rs1922892^***#***^	Intron 13	SLI [12]	**0.039**	0.908	0.794	0.940	0.332 (rs759178)	0.359	0.346
rs2538991^***#***^	Intron 13	SLI [12]	**0.041**	0.852	0.797	0.989	0.332 (rs759178)	0.366	0.338
rs17236239	Intron 13	ASD, SCZ, SLI [12, 23, 27, 46, 49]	0.142	0.290	0.278	0.883	**0.006**	0.622	0.954
rs2538976^***#***^	Intron 13	SLI, SSD [12, 50]	0.051	0.718	0.692	0.812	0.358	0.408	0.424
rs2215798	Intron 13	ASD [26]	0.5	0.469	0.361	0.742	0.030	0.568	0.281
rs4431523	Intron 13	SLI [12]	0.275	0.844	0.676	0.614	**0.001** (rs2708267)	0.933	0.972
rs2710117	Intron 14	SLI, MDD [12, 46, 49]	0.1477	0.6701	0.7566	0.2106	0.894 (rs2710121)	0.993	0.321
rs2710093	Intron 14	ASD [26]	0.4077	0.2891	0.02819	0.2943	0.090 (rs2710091)	0.8416	0.2767

The disease for which association at each listed SNP is given, along with the reference number for each study and the approximate location of each variant within the *CNTNAP2* gene structure. On the right, the *P-value* from each Psychiatric Genomics Consortium (PGC) dataset is reported. Where the associated SNP was not found in the GWAS summary statistic data, results for an alternative SNP are shown in parenthesis (r^2^ = 1). Putative functional SNPs rs7794745 and rs2710102 are underlined. No association survives correction for multiple independent tests (*P* <3.8E-04), but *P-values* < 0.05 are shown in bold. Abbreviations: ASD, autism spectrum disorder; SLI, specific language impairment; DYS, dyslexia; ANX, social anxiety; LAN, language in general population; SCZ, schizophrenia; BD, bipolar disorder; ALD, Alcohol dependence; OPN, Openness general population; MDD, major depressive disorder; SSD, speech sound disorder; N/A, SNP not genotyped

&, r^2^>0.97 across the following SNPs: rs851715 and rs10246256

#, r^2^>0.97 across the following SNPs: rs2710102, rs759178, rs1922892, rs2538991 and rs2538976

^, summary data at this SNP was not included in the latest autism GWAS (PGC2) but was present in the previous data set which included 5,305 ASD cases and 5,305 controls.

### Gene-based analysis of cross-disorder associations

Next, we explored the contribution of common variants across *CNTNAP2* by performing a gene-based association study in MAGMA using GWAS summary statistics from PGC data of seven psychiatric disorders in European populations (Table 3). Association plots for all SNPs included in analysis of each individual phenotype are shown in supporting information (S1 Fig).

**Table 3:** Gene-based tests for association of *CNTNAP2* across seven psychiatric disorders using GWAS summary statistics of the PGC data sets.

Disease	N Cases–Controls	N SNPs tested	Gene-based *P-value*	Top SNP	Top SNP *P-value*
ADHD^a^	19,099–34,194	7,538	0.16	rs370840971	4.8E-04
AN^a^	3,495–10,982	9,318	0.33	rs138287908	8.5E-05
ASD^a^	6,197–7,377	5,946	0.54	rs1089600	0.0018
BD^a^	20,352–31,358	11,345	0.34	rs181471483	3.6E-04
MDD^b^	9,240–9,519	1,214	***0*.*029***	rs4725752	9.3E-04
OCD^a^	2,688–7,037	8,631	0.30	rs6976859	8.7E-05
SCZ^a^	33,640–43,456	12,264	0.11	rs78093069	1.1E-04

The numbers (N) of cases and controls in each dataset examined are given, along with the number of SNPs tested in each dataset. The name and *P-value* of the SNP with most significant association is given. Abbreviations: ADHD, attention-deficit/hyperactivity disorder; AN, anorexia nervosa; ASD, Autism spectrum disorder; BD, bipolar disorder; MDD, major depressive disorder; OCD, obsessive compulsive disorder; SCZ, schizophrenia

^a^, European individuals from the PGC2 data sets

^b^, European individuals from the PGC1 data sets.

The test included a dense coverage of SNPs across *CNTNAP2*: from 1,214 SNPs in MDD up to 12,264 SNPs in schizophrenia. The results suggest that common variants overall do not contribute to disease susceptibility of these phenotypes (Table 3). The most significant association observed was for MDD phase 1 analysis (*P* = 0.029), which is the dataset with the most modest coverage of markers.

To explore whether any gene-based signal is not being detected due to a high signal-to-noise ratio (i.e. inclusion of a large number of SNPs of no functional consequence), we selected 63 predicted functional SNPs in *CNTNAP2* and performed a meta-analysis across psychiatric disorders (for regional association plot, see S2 Fig). Nominal significance of association was observed for 11 predicted functional SNPs with *P-values* ranging from 0.01 and 0.05, but none survive correction for multiple comparisons (Table 4).

**Table 4:** Cross psychiatric disorders meta-analysis of 63 predicted functional SNPs.

SNPs	Allele	Function	Datasets	I	*P-val*	OR
rs17480644	A/G	TFBS	ADHD, AN, BD, OCD, SCZ	8	0.083	1.039
rs1260124	A/T	TFBS	ADHD, AN, ASD, BD, OCD, SCZ	0	0.635	0.996
rs35796336	T/C	TFBS	AN, ASD, BD, OCD, SCZ	0	**0.049**	1.027
rs10277276	T/C	TFBS	BD, OCD, SCZ	0	0.764	0.992
rs34712024	A/G	TFBS	ADHD, AN, BD, OCD, SCZ	32	0.626	1.012
rs2462603	A/G	TFBS	ADHD, AN, ASD, BD, OCD, SCZ	0	0.303	0.993
rs1639484	A/T	SeqCons	ADHD, AN, ASD, BD, OCD, SCZ	0	0.376	1.005
rs12703814	A/G	SeqCons	ADHD, AN, ASD, BD, OCD, SCZ	0	0.965	0.999
rs1639447	A/C	SeqCons	ADHD, AN, ASD, BD, OCD, SCZ	0	0.376	0.990
rs769344	C/G	SeqCons	ADHD, AN, ASD, BD, OCD, SCZ	0	0.778	0.996
rs10280967	A/G	SeqCons	ADHD, AN, ASD, BD, OCD, SCZ	0	0.639	0.996
rs10243142	T/C	SeqCons	ADHD, AN, ASD, BD, OCD, SCZ	0	0.738	1.002
rs12535047	T/C	SeqCons	ADHD, AN, ASD, BD, OCD, SCZ, MDD	0	0.327	0.993
rs347201	A/G	SeqCons	ADHD, AN, ASD, BD, OCD, SCZ	0	0.089	1.011
rs13234249	T/C	SeqCons	ADHD, AN, ASD, BD, OCD, SCZ, MDD	2	0.101	1.011
rs12666908	T/C	SeqCons	ADHD, AN, ASD, BD, OCD, SCZ	0	0.914	0.999
rs11972428	T/G	SeqCons	ADHD, AN, BD, OCD, SCZ	0	0.672	1.012
rs34222835	A/G	SeqCons	ADHD, AN, ASD, BD, OCD, SCZ	4	**0.045**	0.979
rs10261412	A/G	SeqCons	ADHD, AN, ASD, BD, OCD, SCZ, MDD	52	0.808	0.995
rs1826843	A/G	SeqCons	ADHD, AN, ASD, BD, OCD, SCZ, MDD	20	0.153	0.990
rs17170356	A/G	SeqCons	ADHD, AN, BD, OCD, SCZ	0	0.137	0.972
rs4726831	A/C	SeqCons	ADHD, AN, ASD, BD, OCD, SCZ	18	0.294	0.990
rs10279700	T/C	SeqCons	ADHD, AN, ASD, BD, OCD, SCZ, MDD	26	0.857	0.998
rs35701811	A/G	SeqCons	ADHD, AN, BD, OCD, SCZ	0	0.899	0.998
rs899617	T/C	SeqCons	ADHD, AN, ASD, BD, OCD, SCZ	0	**0.026**	1.014
rs747140	C/G	SeqCons	ADHD, AN, ASD, BD, OCD, SCZ	0	**0.025**	1.014
rs7798078	A/G	SeqCons	ADHD, AN, ASD, BD, OCD, SCZ, MDD	0	0.091	1.011
rs34592169	A/G	Splicing	ADHD, AN, ASD, BD, OCD, SCZ	15	0.067	1.014
rs6970064	A/G	SeqCons	ADHD, AN, ASD, BD, OCD, SCZ, MDD	7	0.462	1.004
rs17170640	A/G	SeqCons	ADHD, AN, BD, OCD, SCZ	0	**0.018**	1.041
rs16883690	A/C	SeqCons	BD, SCZ	66	0.673	1.066
rs7797724	T/C	SeqCons	BD, SCZ	0	**0.047**	1.592
rs851659	A/C	SeqCons	ADHD, AN, ASD, BD, OCD, SCZ	9	0.349	0.993
rs35815165	-/AA	SeqCons	ADHD, AN	0	0.831	1.002
rs13247212	T/C	SeqCons	ADHD, AN, ASD, BD, OCD, SCZ	0	0.797	0.996
rs1177007	A/G	SeqCons	ADHD, AN, ASD, BD, OCD, SCZ, MDD	0	0.238	1.008
rs12154883	T/G	SeqCons	ADHD, AN, ASD, BD, OCD, SCZ	0	0.645	1.006
rs13438769	T/C	SeqCons	ADHD, AN, ASD, BD, OCD, SCZ, MDD	0	0.057	1.018
rs2707580	T/G	SeqCons	ADHD, AN, ASD, BD, OCD, SCZ	0	0.146	0.990
rs2707581	T/C	SeqCons	ADHD, AN, ASD, BD, OCD, SCZ	0	0.156	0.990
rs2141955	A/G	SeqCons	ADHD, AN, ASD, BD, OCD, SCZ	0	**0.032**	1.015
rs34347668	A/C	SeqCons	ADHD, AN, ASD, BD, OCD, SCZ	0	0.973	1.000
rs4725756	A/C	SeqCons	ADHD, AN, ASD, BD, OCD, SCZ, MDD	0	**0.015**	0.984
rs2888540	T/C	SeqCons	ADHD, AN, ASD, BD, OCD, SCZ, MDD	0	**0.023**	1.014
rs17170789	A/T	SeqCons	ADHD, AN, BD, OCD, SCZ	0	0.566	1.010
rs17170801	A/C	SeqCons	ADHD, AN, BD, OCD, SCZ	0	0.643	0.989
rs10279343	T/C	SeqCons	ADHD, AN, BD, OCD, SCZ	1	0.568	1.014
rs1122622	A/C	SeqCons	AN, BD, OCD, SCZ	66	0.745	1.026
rs5888312	-/A	SeqCons	ADHD, AN	70	0.744	1.010
rs9648691	A/G	SeqCons, Splicing	ADHD, AN, ASD, BD, OCD, SCZ, MDD	64	0.806	1.002
rs987456	A/C	miRNA	ADHD, AN, ASD, BD, OCD, SCZ, MDD	29	0.570	1.004
rs2717809	C/G	miRNA	AN, BD, OCD, SCZ	0	0.746	0.989
rs2530312	A/G	miRNA	ADHD, AN, ASD, BD, OCD, SCZ	70	0.485	0.990
rs3194	A/C	miRNA	ADHD, AN, ASD, BD, OCD, SCZ, MDD	68	0.797	1.003
rs10243309	C/T	miRNA	AN, MDD	15	0.125	1.149
rs17170999	A/G	miRNA	AN, BD, OCD, SCZ, MDD	0	**0.026**	0.917
rs2530311	A/G	miRNA	ADHD, AN, ASD, BD, OCD, SCZ, MDD	66	0.975	0.999
rs17171000	T/C	miRNA	ADHD, AN, BD, OCD, SCZ, MDD	0	0.583	0.988
rs10251347	C/G	miRNA	ADHD, AN, BD, OCD, SCZ, MDD	0	0.428	0.986
rs2717829	C/G	miRNA	ADHD, AN, ASD, BD, OCD, SCZ, MDD	50	0.820	0.997
rs10280038	A/G	miRNA	AN, BD, OCD, SCZ, MDD	0	**0.031**	1.087
rs2530310	T/C	miRNA	ADHD, AN, ASD, BD, OCD, SCZ, MDD	67	0.666	0.994
rs17171006	T/C	miRNA	ADHD, AN, BD, OCD, SCZ	0	0.493	0.985

For each predicted functional SNP, the alternative alleles and predicted function are listed. *P-values* (*P-val*) were calculated considering fixed-model effect, except SNPs with evidence of heterogeneity (I>50) where odds ratios (OR) were considered under random-effects. Nominally significant associations are indicated in bold *(P-values<0*.*05)*, but none exceed correction for multiple testing (*P*<7.9E-04). Abbreviations: TFBS, transcription factor binding site; SeqCons, sequence conserved nucleotide across species; miRNA, predicted miRNA binding site; Splicing, exonic splicing enhancer (ESE).

The only predicted functional SNP which was previously reported as being associated with ASD was rs34712024 [25], but this variant was not associated with autism in the PGC dataset (*P* = 0.67; Table 2), nor other psychiatric phenotypes examined separately or together (Tables 2–4). MAGMA gene-based association analysis using this more restricted pool of common putative functional variants revealed significant association with ADHD after correction for multiple testing (corrected *P-value* = 0.033) and a nominal association with schizophrenia which did not survive multiple testing correction (S1 Table). However, this signal is reduced to trend level in the cross-disorder meta-analysis for this functional SNP-set (*P-value* = 0.11; S1 Table).

### *De novo* variants in *CNTNAP2*

*De novo* variants in protein-coding genes which are predicted to be functionally damaging are considered to be highly pathogenic and have been extensively explored to implicate genes in psychiatric diseases, especially in ASD and schizophrenia [77]. We explored publicly available sequence data from previous projects in psychiatric disorders to assess the rate of coding *de novo* variants in *CNTNAP2* using two databases (NPdenovo, http://www.wzgenomics.cn/NPdenovo/; and denovo-db, http://denovo-db.gs.washington.edu/denovo-db/). No truncating or missense variants were identified across *CNTNAP2* in 15,539 families (including 2,163 controls), and synonymous variants were reported in only two probands with developmental disorder (Table 5).

**Table 5:** *CNTNAP2 de novo* variants identified across several disease-specific sequencing projects.

Phenotype	N Families	Intronic	Synonymous	Missense
ASD	6,171	106	-	-
SCZ	1,164	-	-	-
EE	647	-	-	-
ID	1,101	-	-	-
DD	4,293	-	2	-
Controls	2,163	13	-	-

The number (N) of families in each dataset examined is given. The full list of *de novo* variants observed is listed in S2 Table. Abbreviations: ASD, autism spectrum disorder; SCZ, schizophrenia; EE, epilepsy; ID, intellectual disability; DD, developmental disability.

### Pathogenic Ultra-Rare Variants (URV) of *CNTNAP2* in ASD and Schizophrenia

Finally, we explored the potential impact of pathogenic ultra-rare variants (URV) in *CNTNAP2* using available sequencing datasets of 4,483 patients with ASD and 6,135 patients with schizophrenia compared with 13,042 controls. We considered only those variants predicted to be pathogenic in both SIFT and Polyphen and which are ultra-rare (MAF<0.0001 in Non-Finnish European population; S3 Table). No difference in the total number of URV was observed between controls and patients with ASD (*P* = 0.11), or schizophrenia (*P* = 0.78) (Table 6).

**Table 6:** Burden analysis of *CNTNAP2* ultra-rare variants (URVs) in ASD and SCZ.

	N Individuals	N Pathogenic URVs	*P-Value*
Controls	13,042	59	
SCZ	6,135	26	0.78
ASD	4,483	29	0.11

The selection of variants included missense variants which are predicted to be pathogenic, truncating variants and canonical splice-site variants. The full list of URVs observed is provided in S3 Table. Abbreviations: SCZ, schizophrenia; ASD, autism spectrum disorder.

### Structural variants affecting *CNTNAP2* amongst psychiatric phenotypes

**Fig 1:**
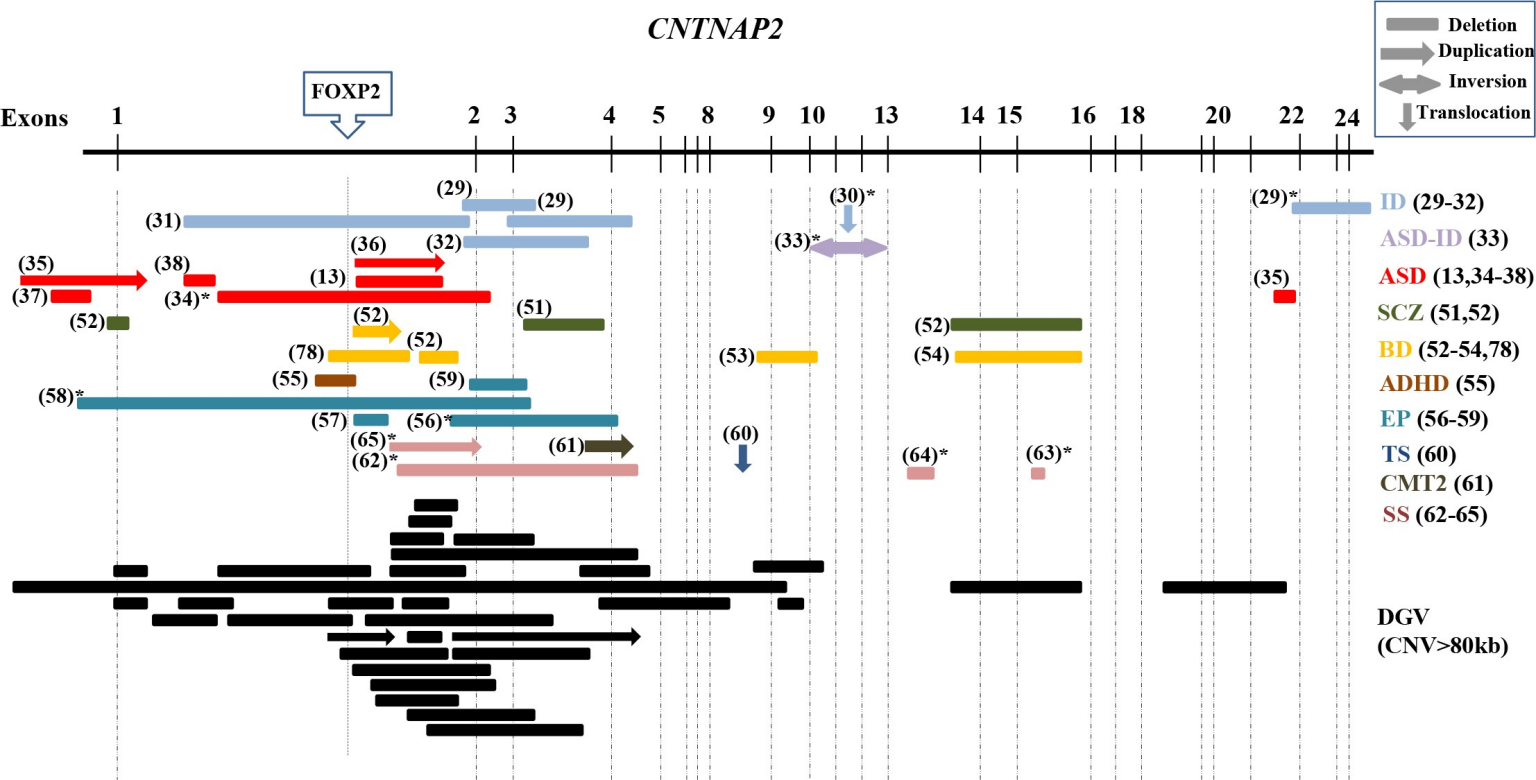
Overview of heterozygous CNVs spanning the *CNTNAP2* gene across several diseases. Abbreviations: ID (Intellectual disability), ASD (autism spectrum disorder), SCZ (schizophrenia), BD (bipolar disorder), ADHD (Attention-deficit/hyperactivity disorder), EP (epilepsy), TS (Tourette syndrome), CMT2 (axonal Charcot-Marie-Tooth), and SS (Speech spectrum: speech delay, childhood apraxia of speech and dyslexia). In parenthesis is reported the reference to each study. *, additional rearrangements reported in this patient. The dashed lines represent the exons and the upper box shows the position of the FOXP2 binding site. In dark shading, CNVs≥80kb found in the general populations from the Database of Genomic Variants are shown.

Several deletions and duplications have been described in neuropsychiatric phenotypes thus far. In Fig 1, we present a comprehensive representation of all previously reported structural variants found in *CNTNAP2* in psychiatric disorders such as ASD or ID [13, 29–38], schizophrenia or bipolar disorder [51–54, 78], ADHD [55], neurologic disorders such as epilepsy, Tourette syndrome or Charcot-Marie-Tooth [56–61]; and finally language-related phenotypes such as speech delay, childhood apraxia of speech and dyslexia [62–65]. Interestingly, the reported structural variants frequently map in intron 1, and extend to exon 4 in some cases. The distribution of those structural variants across different phenotypes does not vary with those found in control populations from the database of genomic variants (http://dgv.tcag.ca/dgv/app/home) (Fig 1), suggesting that structural variants in *CNTNAP2* are not rare events associated exclusively to disease but are present with rare frequency in the general population. Unfortunately, as many reported CNVs come from individual case reports for which the number of subjects screened is not reported, direct frequency comparisons of this data are not meaningful.

### Examination of an intronic deletion in *CNTNAP2* in an extended family with bipolar disorder

**Fig 2:**
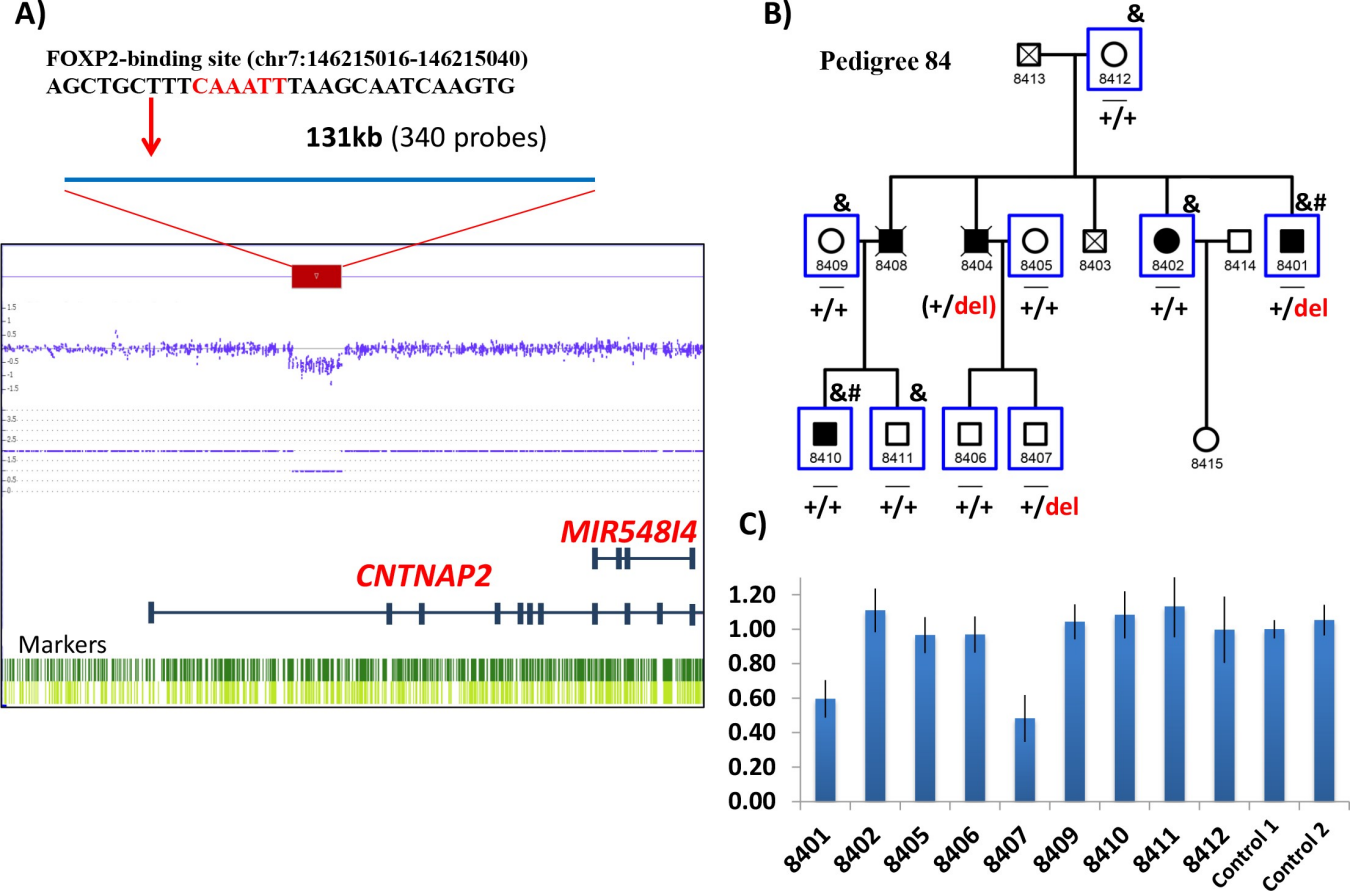
CNV deletion encompassing intron 1 of *CNTNAP2* in an extended family with bipolar disorder. A) CytoScan HD array output image shows the position of the drop in signal intensity of 340 probes, indicating a deletion spanning 131kb (chr7:146203548–146334635; GRCh37/hg19) found in the patient 8401. The position of the FOXP2 binding site within the deletion is shown above. B) The bipolar pedigree includes five patients with bipolar disorder I (BPI) across two generations. Symbols: _, individuals with DNA available; &, individuals with whole exome data; #, individuals analysed for genome-wide CNVs through the CytoScan HD array; blue squares, individuals included in CNV qPCR validation and genotyping analysis, for which heterozygous deletion carriers are indicated as “+/del” and non-carriers are indicated as “+/+”. Inferred genotypes are in parentheses. C) Gene dosage results of the qPCR experiments validating the deletion in patient 8401, and showing the deletion in unaffected subject 8407.

CNV microarray analysis was previously performed in two affected individuals from an extended family which included five relatives affected with bipolar I disorder [78]. A drop in signal intensity for 340 consecutive probes was compatible with a deletion of 131 kb in intron 1 of *CNTNAP2* (hg19/chr7:146203548–146334635; Fig 2A), encompassing the described binding site for the transcription factor FOXP2 (hg19/chr7:146215016–146215040) [12]. The deletion was detected in one of the two affected individuals examined by CNV array. To infer deletion segregation amongst additional relatives, WES-derived genotypes were used to create haplotypes across chromosome 7q35 (Fig 2B). CNV segregation (by haplotype inference) was uninformative due to: 1) incomplete genotype data (unaffected descendants of deceased patient 8404 were not included in the WES study) and 2) a likely recombination at 7q35 in the family. Thus experimental validation and CNV genotyping was performed in all individuals with DNA available to assess the presence of the *CNTNAP2* intronic deletion and its disease association. Using quantitative PCR, the deletion was validated in proband subject 8401, and was detected in one unaffected descendant of deceased patient 8404 (Fig 2B and 2C), implying that: 1) affected subject 8404 would have carried the deletion, had DNA been available; and 2) the CNV is unlikely to be highly penetrant as it was observed in an unaffected adult relative. The structural variant was not detected in the remaining affected relatives and therefore did not segregate with disease status in this family (Fig 2B).

## Discussion

During the last decade, the *CNTNAP2* gene has received considerable attention in the psychiatric genetics field, with a number of studies examining gene dosage, and common or rare variants associations across multiple major psychiatric disorders, which together provided compelling evidence that *CNTNAP2* may be a risk gene with pleiotropic effects in psychiatry. While homozygous mutations in this gene lead to a rare and severe condition described as CASPR2 deficiency disorder (CDD) [7], characterized by profound intellectual disability, epilepsy, language impairment or regression [7, 8], heterozygous mutations or common variants have been suggested to be implicated in autism, whose clinical features overlap with some observed in CDD. *CNTNAP2* is categorised in the SFARI database as syndromic gene and one of the highest-ranking “strong candidate” gene for ASD (https://gene.sfari.org). Heterozygous deletions encompassing the *CNTNAP2* gene were described not only in autism but a wide range of phenotypes, including psychiatric or neurologic disorders, and language-related deficiencies. These structural variants were generally described as causative or highly penetrant [13, 29, 31, 55, 57, 59].

Examination of the distribution of all structural variants described thus far in psychiatric or neurologic patients showed comparable localisation to those found in the general population, suggesting that structural variants affecting *CNTNAP2* may be less relevant in disease susceptibility than previously considered. We were not able to directly compare frequencies of observed structural variants in cases versus controls due to reporting bias in case reports and a lack of information on how many cases were screened to identify those subjects with reportable *CNTNAP2* CNVs, which is a limitation of this study. In the ExAC database, *CNTNAP2* had fewer CNV variants than expected (11 observed vs. 16 expected, z = 0.43; http://exac.broadinstitute.org), and its haploinsufficiency score of 0.59 is in the 8^th^ decile of all genes [79], suggesting that *CNTNAP2* has a tendency to be intolerant to structural variants. A specific case-control CNV analysis is needed to examine CNV frequency differences, but would require a very large sample due to the rarity of CNVs at this locus. A close clinical psychiatric examination of the 66 parents with heterozygous deletions across *CNTNAP2* of CDD provides information on the prevalence of psychiatric conditions in individuals carrying *CNTNAP2* CNVs. All heterozygous family members carrying deletions or truncating mutations were described as phenotypically healthy, suggesting a lack of correlation between these deletions and any major psychiatric condition. Furthermore, parents who were carriers for heterozygous deletions in psychiatric/neurologic patients were described as unaffected at the time of reporting [13, 29, 31, 37, 54, 62], with two exception: one father of a proband with neonatal convulsion, and another father of an epileptic patient, were reported as affected [56, 59]. Moreover, discordant segregation for deletions in *CNTNAP2* was also observed in an ASD sib-pair [13]. Several psychiatric patients who were reported to carry heterozygous structural variants in *CNTNAP2* were also described with translocations or other chromosomal abnormalities [29, 30, 33, 34, 56, 58, 62–65], therefore it is possible that these aberrations may explain the phenotype independently from the observed CNVs in *CNTNAP2*.

We also describe a new CNV deletion which does not segregate with disease in an extended family with bipolar disorder. This CNV removes the FOXP2 transcription factor binding site in intron 1 of *CNTNAP2*, and overlaps with structural variants described in a number of other psychiatric patients. This heterozygous deletion was identified in two individuals with bipolar I disorder from an extended family with five affected members, but was observed also in one unaffected relative (who underwent diagnostic interview at age >40 and therefore was beyond the typical age of symptom onset). Hence, the deletion was not segregating with the disease and is unlikely to represent a highly penetrant risk variant in this family, although we cannot exclude a multiple hit model where the CNV deletion interacts with other etiologic risk variants at other loci to exert phenotypic effect.

*CNTNAP2*^*-/-*^ knock-out mice have been proposed as valid animal model for ASD considering the phenotypic similarities between ASD and the CASPR2 deficiency disorder [2]. *CNTNAP2*^*-/-*^ knock-out mice showed abnormalities in the arborisation of dendrites, maturation of dendritic spines, defects in migration of cortical projection neurons, and reduction of GABAergic interneurons [2, 4]. Controversially, ASD is not a core feature in the most recent patient series reported with CASPR2 deficiency disorder [7, 8]. The association previously proposed around the relationship between heterozygous deletions in *CNTNAP2* and ASD does not have a support from mouse models, as heterozygous mice did not show any behavioural or neuropathological abnormalities that were observed in homozygous knockouts [2]. Notwithstanding this, it is possible that the combination of heterozygous *CNTNAP2* deletions in a genomic background of increased risk (through inheritance of other common and rare risk variants at other loci) may lead to psychiatric, behavioural or neuropathological abnormalities.

Common variants in *CNTNAP2* are another class of genetic variation associated with several psychiatric or language-related phenotypes. The most interesting finding from studies of this variant class converge on markers rs7794745 and rs2710102, originally reported in ASD [13, 14], and replicated later in ASD or implicated in other phenotypes [12, 15, 23, 24, 46–48]. Neuroimaging studies have supported the notion that these common variants play a role in psychiatric disorders. SNP rs2710102 has been implicated in brain connectivity in healthy individuals [16, 18, 19], and rs7794745 was implicated in audio-visual speech perception [80], voice-specific brain function [22], and was associated with reduced grey matter volume in left superior occipital gyrus [20, 21]. These studies focused principally on language tasks in general population, given the reported suggestive implications of *CNTNAP2* in language impairment traits of ASD or language-related phenotypes. However, the direct role of *CNTNAP2* in language is still unclear; indeed the language regression observed in patients with CASPR2 deficiency disorder are concomitant with seizure onset and may represent a secondary phenotypic effect caused by seizures [7]. On the other hand, the first genetic association of rs7794745 and rs2710102 with ASD, as well as the other psychiatric diseases were based in studies with limited sample size, and recent studies failed to replicate associations between the two markers and ASD [81, 82]. Individual alleles associated in the past with limited numbers of patients warrant replications in adequately powered samples to ascertain *bona fide* findings considering the small size effects of common variants [83], such as that attempted here. Using the largest case-control cohorts currently publicly available (PGC datasets), we did not find evidence for significant association of previously reported common variants, or a combined effect for common variants of *CNTNAP2* in the susceptibility of psychiatric disorders, nor did we find predicted functional SNPs with a role across disorders.

Finally, we examine evidence for rare variant contributions in *CNTNAP2*. Rare variants in the promoter or coding region were reported to play a role in the pathophysiology of ASD [25, 33], although a recent study including a large number of cases and controls did not find association of rare variants of *CNTNAP2* in ASD [84]. Here we report the largest sample investigated thus far in ASD and schizophrenia, which suggests that rare variants in *CNTNAP2* do not play a major role in these two psychiatric disorders. Furthermore, examination of *de novo* variants in combined psychiatric sequencing projects of over 15,500 trios suggest that *de novo* variants in *CNTNAP2* do not increase risk for psychiatric disorders.

While functional studies show a relationship between certain deletions or rare variants of *CNTNAP2* with neuronal phenotypes relevant to psychiatric illness [25, 54, 85], we show that the genetic link between these variants and psychiatric phenotypes is tenuous. However, this does not dispel the evidence that the *CNTNAP2* gene, or specific genetic variations within this gene, may have a real impact on neuronal functions or variability of brain connectivity in the general population.

It is now possible to combine large datasets to ascertain the real impact of candidate genes described in the past in psychiatric disorders. Here we performed analyses using large publicly available datasets investigating a range of mutational mechanisms which impact variability of *CNTNAP2* across several psychiatric disorders. In conclusion, our results converge to show a limited or likely neutral role of *CNTNAP2* in the susceptibility of psychiatric disorders. However, the impact of this gene in language deficit *per se* is not directly examined in this study and warrants additional investigation.

## Methods

### Common variant association in *CNTNAP2* using publicly available datasets

We sought to replicate previously reported *CNTNAP2* SNP associations in a range of psychiatric phenotypes or traits using GWAS summary-statistic data of the Psychiatric Genomics Consortium (https://med.unc.edu/pgc/results-and-downloads).

Firstly, we report the corresponding *P-value*s of specific previously associated markers for case-control cohorts with autism spectrum disorder (ASD), schizophrenia (SCZ), bipolar disorder (BD), attention-deficit hyperactivity-disorder (ADHD), major depressive disorder (MDD), anorexia nervosa (AN), and obsessive compulsive disorder (OCD). If a specific SNP marker was not reported in an individual GWAS dataset, we selected another marker in high linkage disequilibrium (r^2^~1, using genotype data from the CEU, TSI, GBR and IBS European populations in 1000genomes project; http://www.internationalgenome.org).

Next, a gene-based association for common variants was calculated with MAGMA [86], using variants within a 5 kb window upstream and downstream of *CNTNAP2*. Selected datasets were of European descent, derived from GWAS summary statistics of the Psychiatric Genomics Consortium (https://med.unc.edu/pgc/results-and-downloads): SCZ (33,640 cases and 43,456 controls), BD (20,352 cases and 31,358 controls), ASD (6,197 and 7,377 controls), ADHD (19,099 cases and 34,194 controls), MDD (9,240 cases and 9,519 controls), OCD (2,688 cases and 7,037 controls), and AN (3,495 cases and 10,982 controls) [87–93]. Analyses were performed combining two different models for higher statistical power and sensitivity when the genetic architecture is unknown: the combined *P-value* model, which is more sensitive when only a small proportion of key SNPs in a gene show association; and the mean SNP association, which is more sensitive when allelic heterogeneity is greater and a larger number of SNPs show nominal association.

Finally, we selected SNPs predicted to be functional within a 5kb window upstream/downstream of *CNTNAP2* (e.g. located in transcription factor binding sites, miRNA binding sites etc; https://snpinfo.niehs.nih.gov), and assessed a potential cross-disorder effect using GWAS summary statistics data of the PGC by performing a meta-analysis in PLINK [94]. The Cochran’s Q-statistic and I^2^ statistic were calculated to examine heterogeneity amongst studies. The null hypothesis was that all studies were measuring the same true effect, which would be rejected if heterogeneity exists across studies. For all functional SNPs, when heterogeneity between studies was I>50% (*P*<0.05), the pooled OR was estimated using a random-effects model.

### Analysis of rare variants in *CNTNAP2* in ASD and schizophrenia, and *de novo* variants across psychiatric cohorts

The impact of rare variants of *CNTNAP2* was assessed using sequencing-level data from the following datasets: WES from the Sweden-Schizophrenia population-based Case-Control cohort (6,135 cases and 6,245 controls; dbGAP accession: phs000473.v2.p2); ARRA Autism Sequencing Collaboration (490 BCM cases, BCM 486 controls, and 1,288 unrelated ASD probands from consent code c1; dbGAP accession: phs000298.v3.p2); Medical Genome Reference Bank (2,845 healthy Australian adults; https://sgc.garvan.org.au/initiatives/mgrb); individuals from a Caucasian Spanish population (719 controls [95, 96]); in-house ASD patients (30 cases; [97]); and previous published dataset in ASD (2,704 cases and 2,747 controls [84]). The selection of potentially etiologic variants was performed based on their predicted pathogenicity (missense damaging in both SIFT and polyphen 2, canonical splice variants, stop mutation and indels) and minor allele frequency (MAF<0.0001 in non-Finnish European populations using the Genome Aggregation Database; http://gnomad.broadinstitute.org/). A chi square statistic was used to compare separately the sample of schizophrenia patients (6,135 cases) and the combined ASD datasets (4,512 cases) with the combined control datasets (13,042 individuals).

Two databases for *de novo* variants were used to identify *de novo* variants in *CNTNAP2* [98, 99], which comprise data for the following samples: autism spectrum disorder (6,171 families), schizophrenia (1,164 families), epilepsy (647 families), intellectual disability (1,101 families), developmental disorders (4,293 families) and controls (2,163).

### Extended family with bipolar disorder and CNV in *CNTNAP2*

The extended family presented here (Fig 2B) provides a molecular follow-up from a previously reported whole exome sequencing (WES) study of multiplex BD families, augmented with CNV microarray data [78]. This multigenerational pedigree, was collected through the Mood Disorders Unit and Black Dog Institute at the Prince of Wales Hospital, Sydney, and the School of Psychiatry (University of New South Wales in Sydney) [100–104]. Consenting family members were assessed using the Family Interview for Genetic Studies (FIGS) [105], and the Diagnostic Interview for Genetic Studies (DIGS) [106]. The study was approved by the Human Research Ethics Committee of the University of New South Wales, and written informed consent was obtained from all participating individuals. Blood samples were collected for DNA extraction by standard laboratory methods. Three of the five relatives with bipolar disorder type I (BD-I) had DNA and WES-derived genotype data available, and six unaffected relatives with DNA and WES data were available for haplotype phasing and segregation analysis (Fig 2B).

Genome-wide CNV analysis was performed via CytoScan HD Array (Affymetrix, Santa Clara, CA, USA) in 2 distal affected relatives (individuals 8410 and 8401; Fig 2B), using the Affymetrix Chromosome Analysis Suite (ChAS) software (ThermoFisher, Waltham, MA, USA). Detailed information on CNV detection and filtering criteria have been previously described [78]. We identified a 131kb deletion in intron 1 of *CNTNAP2* in individual 8401. WES-derived genotypes were used for haplotype assessment to infer CNV segregation amongst relatives, as previously described [78]. Next, we experimentally validated the *CNTNAP2* CNV via quantitative PCR (qPCR) in all available family members. Validation was performed in quadruplicate via a SYBR Green-based quantitative PCR (qPCR) method using two independent amplicon probes, each compared with two different reference amplicon probes in the *FOXP2* and *RNF20* genes (S4 Table). Experimental details are available upon request.

## Supporting information

S1 Fig CNTNAP association plots using GWAS summary statistics of the PGC data sets. (DOCX)

S2 Fig Cross-disorder association plot of *CNTNAP2* common variants predicted to be functional (63 SNPs). (DOCX)

S1 Table Gene-based analysis of predicted functional SNPs across seven psychiatric disorders. (DOCX)

S2 Table Full list of d*e novo* variants in *CNTNAP2* gene. (DOCX)

S3 Table Full list of Ultra-Rare Variants (URVs) in available sequencing datasets. (DOCX)

S4 Table Primers used in the CNV validation for the *CNTNAP2* intronic deletion. (DOCX)

## References

[1] Poliak S, Salomon D, Elhanany H, Sabanay H, Kiernan B, Pevny L, et al Juxtaparanodal clustering of Shaker-like K+ channels in myelinated axons depends on Caspr2 and TAG-1. The Journal of cell biology. 2003;162(6):1149–60. 10.1083/jcb.200305018 12963709 PMC2172860

[2] Penagarikano O, Abrahams BS, Herman EI, Winden KD, Gdalyahu A, Dong H, et al Absence of CNTNAP2 leads to epilepsy, neuronal migration abnormalities, and core autism-related deficits. Cell. 2011;147(1):235–46. 10.1016/j.cell.2011.08.040 21962519 PMC3390029

[3] Abrahams BS, Tentler D, Perederiy JV, Oldham MC, Coppola G, Geschwind DH. Genome-wide analyses of human perisylvian cerebral cortical patterning. Proceedings of the National Academy of Sciences of the United States of America. 2007;104(45):17849–54. 10.1073/pnas.0706128104 17978184 PMC2077018

[4] Anderson GR, Galfin T, Xu W, Aoto J, Malenka RC, Sudhof TC. Candidate autism gene screen identifies critical role for cell-adhesion molecule CASPR2 in dendritic arborization and spine development. Proceedings of the National Academy of Sciences of the United States of America. 2012;109(44):18120–5. 10.1073/pnas.1216398109 23074245 PMC3497786

[5] Strauss KA, Puffenberger EG, Huentelman MJ, Gottlieb S, Dobrin SE, Parod JM, et al Recessive symptomatic focal epilepsy and mutant contactin-associated protein-like 2. The New England journal of medicine. 2006;354(13):1370–7. 10.1056/NEJMoa052773 . 16571880

[6] Zweier C, de Jong EK, Zweier M, Orrico A, Ousager LB, Collins AL, et al CNTNAP2 and NRXN1 are mutated in autosomal-recessive Pitt-Hopkins-like mental retardation and determine the level of a common synaptic protein in Drosophila. American journal of human genetics. 2009;85(5):655–66. 10.1016/j.ajhg.2009.10.004 19896112 PMC2775834

[7] Rodenas-Cuadrado P, Pietrafusa N, Francavilla T, La Neve A, Striano P, Vernes SC. Characterisation of CASPR2 deficiency disorder—a syndrome involving autism, epilepsy and language impairment. BMC medical genetics. 2016;17:8 10.1186/s12881-016-0272-8 26843181 PMC4739328

[8] Smogavec M, Cleall A, Hoyer J, Lederer D, Nassogne MC, Palmer EE, et al Eight further individuals with intellectual disability and epilepsy carrying bi-allelic CNTNAP2 aberrations allow delineation of the mutational and phenotypic spectrum. Journal of medical genetics. 2016;53(12):820–7. 10.1136/jmedgenet-2016-103880 . 27439707

[9] Watson CM, Crinnion LA, Tzika A, Mills A, Coates A, Pendlebury M, et al Diagnostic whole genome sequencing and split-read mapping for nucleotide resolution breakpoint identification in CNTNAP2 deficiency syndrome. American journal of medical genetics Part A. 2014;164A(10):2649–55. 10.1002/ajmg.a.36679 . 25045150

[10] Kale T, Philip M. An Interstitial Deletion at 7q33-36.1 in a Patient with Intellectual Disability, Significant Language Delay, and Severe Microcephaly. Case reports in genetics. 2016;2016:6046351 10.1155/2016/6046351 28053794 PMC5178345

[11] Lai CS, Fisher SE, Hurst JA, Vargha-Khadem F, Monaco AP. A forkhead-domain gene is mutated in a severe speech and language disorder. Nature. 2001;413(6855):519–23. 10.1038/35097076 . 11586359

[12] Vernes SC, Newbury DF, Abrahams BS, Winchester L, Nicod J, Groszer M, et al A functional genetic link between distinct developmental language disorders. The New England journal of medicine. 2008;359(22):2337–45. 10.1056/NEJMoa0802828 18987363 PMC2756409

[13] Alarcon M, Abrahams BS, Stone JL, Duvall JA, Perederiy JV, Bomar JM, et al Linkage, association, and gene-expression analyses identify CNTNAP2 as an autism-susceptibility gene. American journal of human genetics. 2008;82(1):150–9. 10.1016/j.ajhg.2007.09.005 18179893 PMC2253955

[14] Arking DE, Cutler DJ, Brune CW, Teslovich TM, West K, Ikeda M, et al A common genetic variant in the neurexin superfamily member CNTNAP2 increases familial risk of autism. American journal of human genetics. 2008;82(1):160–4. 10.1016/j.ajhg.2007.09.015 18179894 PMC2253968

[15] Whitehouse AJ, Bishop DV, Ang QW, Pennell CE, Fisher SE. CNTNAP2 variants affect early language development in the general population. Genes, brain, and behavior. 2011;10(4):451–6. 10.1111/j.1601-183X.2011.00684.x 21310003 PMC3130139

[16] Whalley HC, O'Connell G, Sussmann JE, Peel A, Stanfield AC, Hayiou-Thomas ME, et al Genetic variation in CNTNAP2 alters brain function during linguistic processing in healthy individuals. American journal of medical genetics Part B, Neuropsychiatric genetics: the official publication of the International Society of Psychiatric Genetics. 2011;156B(8):941–8. 10.1002/ajmg.b.31241 . 21987501

[17] Clemm von Hohenberg C, Wigand MC, Kubicki M, Leicht G, Giegling I, Karch S, et al CNTNAP2 polymorphisms and structural brain connectivity: a diffusion-tensor imaging study. Journal of psychiatric research. 2013;47(10):1349–56. 10.1016/j.jpsychires.2013.07.002 23871450 PMC3780783

[18] Scott-Van Zeeland AA, Abrahams BS, Alvarez-Retuerto AI, Sonnenblick LI, Rudie JD, Ghahremani D, et al Altered functional connectivity in frontal lobe circuits is associated with variation in the autism risk gene CNTNAP2. Science translational medicine. 2010;2(56):56ra80 10.1126/scitranslmed.3001344 21048216 PMC3065863

[19] Dennis EL, Jahanshad N, Rudie JD, Brown JA, Johnson K, McMahon KL, et al Altered structural brain connectivity in healthy carriers of the autism risk gene, CNTNAP2. Brain Connect. 2011;1(6):447–59. 10.1089/brain.2011.0064 22500773 PMC3420970

[20] Tan GC, Doke TF, Ashburner J, Wood NW, Frackowiak RS. Normal variation in fronto-occipital circuitry and cerebellar structure with an autism-associated polymorphism of CNTNAP2. NeuroImage. 2010;53(3):1030–42. 10.1016/j.neuroimage.2010.02.018 20176116 PMC2941042

[21] Udden J, Snijders TM, Fisher SE, Hagoort P. A common variant of the CNTNAP2 gene is associated with structural variation in the left superior occipital gyrus. Brain and language. 2017;172:16–21. 10.1016/j.bandl.2016.02.003 . 27059522

[22] Koeda M, Watanabe A, Tsuda K, Matsumoto M, Ikeda Y, Kim W, et al Interaction effect between handedness and CNTNAP2 polymorphism (rs7794745 genotype) on voice-specific frontotemporal activity in healthy individuals: an fMRI study. Frontiers in behavioral neuroscience. 2015;9:87 10.3389/fnbeh.2015.00087 25941478 PMC4403548

[23] Steer CD, Golding J, Bolton PF. Traits contributing to the autistic spectrum. PloS one. 2010;5(9):e12633 10.1371/journal.pone.0012633 20838614 PMC2935882

[24] Nascimento PP, Bossolani-Martins AL, Rosan DB, Mattos LC, Brandao-Mattos C, Fett-Conte AC. Single nucleotide polymorphisms in the CNTNAP2 gene in Brazilian patients with autistic spectrum disorder. Genetics and molecular research: GMR. 2016;15(1). 10.4238/gmr.15017422 . 26909962

[25] Chiocchetti AG, Kopp M, Waltes R, Haslinger D, Duketis E, Jarczok TA, et al Variants of the CNTNAP2 5' promoter as risk factors for autism spectrum disorders: a genetic and functional approach. Molecular psychiatry. 2015;20(7):839–49. 10.1038/mp.2014.103 . 25224256

[26] Sampath S, Bhat S, Gupta S, O'Connor A, West AB, Arking DE, et al Defining the contribution of CNTNAP2 to autism susceptibility. PloS one. 2013;8(10):e77906 10.1371/journal.pone.0077906 24147096 PMC3798378

[27] Anney R, Klei L, Pinto D, Almeida J, Bacchelli E, Baird G, et al Individual common variants exert weak effects on the risk for autism spectrum disorders. Human molecular genetics. 2012;21(21):4781–92. 10.1093/hmg/dds301 22843504 PMC3471395

[28] Poot M. A candidate gene association study further corroborates involvement of contactin genes in autism. Molecular syndromology. 2014;5(5):229–35. 10.1159/000362891 25337070 PMC4188154

[29] Gregor A, Albrecht B, Bader I, Bijlsma EK, Ekici AB, Engels H, et al Expanding the clinical spectrum associated with defects in CNTNAP2 and NRXN1. BMC medical genetics. 2011;12:106 10.1186/1471-2350-12-106 21827697 PMC3162517

[30] Belloso JM, Bache I, Guitart M, Caballin MR, Halgren C, Kirchhoff M, et al Disruption of the CNTNAP2 gene in a t(7;15) translocation family without symptoms of Gilles de la Tourette syndrome. European journal of human genetics: EJHG. 2007;15(6):711–3. 10.1038/sj.ejhg.5201824 . 17392702

[31] Polimanti R, Squitti R, Pantaleo M, Giglio S, Zito G. Duplication of FOXP2 binding sites within CNTNAP2 gene in a girl with neurodevelopmental delay. Minerva pediatrica. 2017;69(2):162–4. 10.23736/S0026-4946.16.04326-7 . 27215640

[32] Mikhail FM, Lose EJ, Robin NH, Descartes MD, Rutledge KD, Rutledge SL, et al Clinically relevant single gene or intragenic deletions encompassing critical neurodevelopmental genes in patients with developmental delay, mental retardation, and/or autism spectrum disorders. American journal of medical genetics Part A. 2011;155A(10):2386–96. 10.1002/ajmg.a.34177 . 22031302

[33] Bakkaloglu B, O'Roak BJ, Louvi A, Gupta AR, Abelson JF, Morgan TM, et al Molecular cytogenetic analysis and resequencing of contactin associated protein-like 2 in autism spectrum disorders. American journal of human genetics. 2008;82(1):165–73. 10.1016/j.ajhg.2007.09.017 18179895 PMC2253974

[34] Poot M, Beyer V, Schwaab I, Damatova N, Van't Slot R, Prothero J, et al Disruption of CNTNAP2 and additional structural genome changes in a boy with speech delay and autism spectrum disorder. Neurogenetics. 2010;11(1):81–9. 10.1007/s10048-009-0205-1 . 19582487

[35] Girirajan S, Dennis MY, Baker C, Malig M, Coe BP, Campbell CD, et al Refinement and discovery of new hotspots of copy-number variation associated with autism spectrum disorder. American journal of human genetics. 2013;92(2):221–37. 10.1016/j.ajhg.2012.12.016 23375656 PMC3567267

[36] Prasad A, Merico D, Thiruvahindrapuram B, Wei J, Lionel AC, Sato D, et al A discovery resource of rare copy number variations in individuals with autism spectrum disorder. G3. 2012;2(12):1665–85. 10.1534/g3.112.004689 23275889 PMC3516488

[37] Nord AS, Roeb W, Dickel DE, Walsh T, Kusenda M, O'Connor KL, et al Reduced transcript expression of genes affected by inherited and de novo CNVs in autism. European journal of human genetics: EJHG. 2011;19(6):727–31. 10.1038/ejhg.2011.24 21448237 PMC3110052

[38] Egger G, Roetzer KM, Noor A, Lionel AC, Mahmood H, Schwarzbraun T, et al Identification of risk genes for autism spectrum disorder through copy number variation analysis in Austrian families. Neurogenetics. 2014;15(2):117–27. 10.1007/s10048-014-0394-0 . 24643514

[39] Vogt D, Cho KKA, Shelton SM, Paul A, Huang ZJ, Sohal VS, et al Mouse Cntnap2 and Human CNTNAP2 ASD Alleles Cell Autonomously Regulate PV+ Cortical Interneurons. Cerebral cortex. 2017:1–12. 10.1093/cercor/bhx248 . 29028946 PMC6455910

[40] Scott R, Sanchez-Aguilera A, van Elst K, Lim L, Dehorter N, Bae SE, et al Loss of Cntnap2 Causes Axonal Excitability Deficits, Developmental Delay in Cortical Myelination, and Abnormal Stereotyped Motor Behavior. Cerebral cortex. 2017 10.1093/cercor/bhx341 . 29300891

[41] Wang KS, Liu XF, Aragam N. A genome-wide meta-analysis identifies novel loci associated with schizophrenia and bipolar disorder. Schizophrenia research. 2010;124(1–3):192–9. 10.1016/j.schres.2010.09.002 . 20889312

[42] Zhong X, Zhang H. Linkage analysis and association analysis in the presence of linkage using age at onset of COGA alcoholism data. BMC genetics. 2005;6 Suppl 1:S31 10.1186/1471-2156-6-S1-S31 16451641 PMC1866754

[43] Terracciano A, Sanna S, Uda M, Deiana B, Usala G, Busonero F, et al Genome-wide association scan for five major dimensions of personality. Molecular psychiatry. 2010;15(6):647–56. 10.1038/mp.2008.113 18957941 PMC2874623

[44] Wray NR, Pergadia ML, Blackwood DH, Penninx BW, Gordon SD, Nyholt DR, et al Genome-wide association study of major depressive disorder: new results, meta-analysis, and lessons learned. Molecular psychiatry. 2012;17(1):36–48. 10.1038/mp.2010.109 21042317 PMC3252611

[45] Chen X, Long F, Cai B, Chen X, Chen G. A novel relationship for schizophrenia, bipolar and major depressive disorder Part 7: A hint from chromosome 7 high density association screen. Behavioural brain research. 2015;293:241–51. 10.1016/j.bbr.2015.06.043 . 26192912

[46] Newbury DF, Paracchini S, Scerri TS, Winchester L, Addis L, Richardson AJ, et al Investigation of dyslexia and SLI risk variants in reading- and language-impaired subjects. Behavior genetics. 2011;41(1):90–104. 10.1007/s10519-010-9424-3 21165691 PMC3029677

[47] Peter B, Raskind WH, Matsushita M, Lisowski M, Vu T, Berninger VW, et al Replication of CNTNAP2 association with nonword repetition and support for FOXP2 association with timed reading and motor activities in a dyslexia family sample. Journal of neurodevelopmental disorders. 2011;3(1):39–49. 10.1007/s11689-010-9065-0 21484596 PMC3163991

[48] Stein MB, Yang BZ, Chavira DA, Hitchcock CA, Sung SC, Shipon-Blum E, et al A common genetic variant in the neurexin superfamily member CNTNAP2 is associated with increased risk for selective mutism and social anxiety-related traits. Biological psychiatry. 2011;69(9):825–31. 10.1016/j.biopsych.2010.11.008 21193173 PMC3079072

[49] Ji W, Li T, Pan Y, Tao H, Ju K, Wen Z, et al CNTNAP2 is significantly associated with schizophrenia and major depression in the Han Chinese population. Psychiatry research. 2013;207(3):225–8. 10.1016/j.psychres.2012.09.024 . 23123147

[50] Zhao YJ, Wang YP, Yang WZ, Sun HW, Ma HW, Zhao YR. CNTNAP2 Is Significantly Associated With Speech Sound Disorder in the Chinese Han Population. Journal of child neurology. 2015;30(13):1806–11. 10.1177/0883073815581609 . 25895914

[51] Friedman JI, Vrijenhoek T, Markx S, Janssen IM, van der Vliet WA, Faas BH, et al CNTNAP2 gene dosage variation is associated with schizophrenia and epilepsy. Molecular psychiatry. 2008;13(3):261–6. 10.1038/sj.mp.4002049 . 17646849

[52] Malhotra D, McCarthy S, Michaelson JJ, Vacic V, Burdick KE, Yoon S, et al High frequencies of de novo CNVs in bipolar disorder and schizophrenia. Neuron. 2011;72(6):951–63. 10.1016/j.neuron.2011.11.007 22196331 PMC3921625

[53] Zhang D, Cheng L, Qian Y, Alliey-Rodriguez N, Kelsoe JR, Greenwood T, et al Singleton deletions throughout the genome increase risk of bipolar disorder. Molecular psychiatry. 2009;14(4):376–80. 10.1038/mp.2008.144 19114987 PMC2735188

[54] Lee IS, Carvalho CM, Douvaras P, Ho SM, Hartley BJ, Zuccherato LW, et al Characterization of molecular and cellular phenotypes associated with a heterozygous CNTNAP2 deletion using patient-derived hiPSC neural cells. NPJ schizophrenia. 2015;1 10.1038/npjschz.2015.19 26985448 PMC4789165

[55] Elia J, Gai X, Xie HM, Perin JC, Geiger E, Glessner JT, et al Rare structural variants found in attention-deficit hyperactivity disorder are preferentially associated with neurodevelopmental genes. Molecular psychiatry. 2010;15(6):637–46. 10.1038/mp.2009.57 19546859 PMC2877197

[56] Mefford HC, Muhle H, Ostertag P, von Spiczak S, Buysse K, Baker C, et al Genome-wide copy number variation in epilepsy: novel susceptibility loci in idiopathic generalized and focal epilepsies. PLoS genetics. 2010;6(5):e1000962 10.1371/journal.pgen.1000962 20502679 PMC2873910

[57] Lesca G, Rudolf G, Labalme A, Hirsch E, Arzimanoglou A, Genton P, et al Epileptic encephalopathies of the Landau-Kleffner and continuous spike and waves during slow-wave sleep types: genomic dissection makes the link with autism. Epilepsia. 2012;53(9):1526–38. 10.1111/j.1528-1167.2012.03559.x . 22738016

[58] Curran S, Ahn JW, Grayton H, Collier DA, Ogilvie CM. NRXN1 deletions identified by array comparative genome hybridisation in a clinical case series—further understanding of the relevance of NRXN1 to neurodevelopmental disorders. Journal of molecular psychiatry. 2013;1(1):4 10.1186/2049-9256-1-4 25408897 PMC4223877

[59] Pippucci T, Licchetta L, Baldassari S, Palombo F, Menghi V, D'Aurizio R, et al Epilepsy with auditory features: A heterogeneous clinico-molecular disease. Neurology Genetics. 2015;1(1):e5 10.1212/NXG.0000000000000005 27066544 PMC4821078

[60] Verkerk AJ, Mathews CA, Joosse M, Eussen BH, Heutink P, Oostra BA, et al CNTNAP2 is disrupted in a family with Gilles de la Tourette syndrome and obsessive compulsive disorder. Genomics. 2003;82(1):1–9. . 12809671 10.1016/s0888-7543(03)00097-1

[61] Hoyer H, Braathen GJ, Eek AK, Nordang GB, Skjelbred CF, Russell MB. Copy number variations in a population-based study of Charcot-Marie-Tooth disease. BioMed research international. 2015;2015:960404 10.1155/2015/960404 25648254 PMC4306395

[62] Al-Murrani A, Ashton F, Aftimos S, George AM, Love DR. Amino-Terminal Microdeletion within the CNTNAP2 Gene Associated with Variable Expressivity of Speech Delay. Case reports in genetics. 2012;2012:172408 10.1155/2012/172408 23074684 PMC3447220

[63] Centanni TM, Sanmann JN, Green JR, Iuzzini-Seigel J, Bartlett C, Sanger WG, et al The role of candidate-gene CNTNAP2 in childhood apraxia of speech and specific language impairment. American journal of medical genetics Part B, Neuropsychiatric genetics: the official publication of the International Society of Psychiatric Genetics. 2015;168(7):536–43. 10.1002/ajmg.b.32325 . 26097074

[64] Laffin JJ, Raca G, Jackson CA, Strand EA, Jakielski KJ, Shriberg LD. Novel candidate genes and regions for childhood apraxia of speech identified by array comparative genomic hybridization. Genetics in medicine: official journal of the American College of Medical Genetics. 2012;14(11):928–36. 10.1038/gim.2012.72 22766611 PMC3563158

[65] Veerappa AM, Saldanha M, Padakannaya P, Ramachandra NB. Family-based genome-wide copy number scan identifies five new genes of dyslexia involved in dendritic spinal plasticity. Journal of human genetics. 2013;58(8):539–47. 10.1038/jhg.2013.47 . 23677055

[66] Chang YT, Chen PC, Tsai IJ, Sung FC, Chin ZN, Kuo HT, et al Bidirectional relation between schizophrenia and epilepsy: a population-based retrospective cohort study. Epilepsia. 2011;52(11):2036–42. 10.1111/j.1528-1167.2011.03268.x . 21929680

[67] Chang HJ, Liao CC, Hu CJ, Shen WW, Chen TL. Psychiatric disorders after epilepsy diagnosis: a population-based retrospective cohort study. PloS one. 2013;8(4):e59999 10.1371/journal.pone.0059999 23577079 PMC3618118

[68] Sucksdorff D, Brown AS, Chudal R, Jokiranta-Olkoniemi E, Leivonen S, Suominen A, et al Parental and comorbid epilepsy in persons with bipolar disorder. Journal of affective disorders. 2015;188:107–11. 10.1016/j.jad.2015.08.051 26356289 PMC4631649

[69] Wiglusz MS, Landowski J, Cubala WJ, Agius M. Overlapping phenomena of bipolar disorder and epilepsy—a common pharmacological pathway. Psychiatria Danubina. 2015;27 Suppl 1:S177–81. . 26417756

[70] Tuchman R, Rapin I. Epilepsy in autism. The Lancet Neurology. 2002;1(6):352–8. . 12849396 10.1016/s1474-4422(02)00160-6

[71] Besag FM. Epilepsy in patients with autism: links, risks and treatment challenges. Neuropsychiatric disease and treatment. 2018;14:1–10. 10.2147/NDT.S120509 29296085 PMC5739118

[72] Wells R, Swaminathan V, Sundram S, Weinberg D, Bruggemann J, Jacomb I, et al The impact of premorbid and current intellect in schizophrenia: cognitive, symptom, and functional outcomes. NPJ schizophrenia. 2015;1:15043 10.1038/npjschz.2015.43 27336046 PMC4849463

[73] Pawelczyk A, Kotlicka-Antczak M, Lojek E, Ruszpel A, Pawelczyk T. Schizophrenia patients have higher-order language and extralinguistic impairments. Schizophrenia research. 2018;192:274–80. 10.1016/j.schres.2017.04.030 . 28438437

[74] Raucher-Chene D, Achim AM, Kaladjian A, Besche-Richard C. Verbal fluency in bipolar disorders: A systematic review and meta-analysis. Journal of affective disorders. 2017;207:359–66. 10.1016/j.jad.2016.09.039 . 27744224

[75] Rapin I, Dunn M. Update on the language disorders of individuals on the autistic spectrum. Brain & development. 2003;25(3):166–72. . 12689694 10.1016/s0387-7604(02)00191-2

[76] Tager-Flusberg H, Calkins S, Nolin T, Baumberger T, Anderson M, Chadwick-Dias A. A longitudinal study of language acquisition in autistic and Down syndrome children. Journal of autism and developmental disorders. 1990;20(1):1–21. . 2139024 10.1007/BF02206853

[77] Fromer M, Pocklington AJ, Kavanagh DH, Williams HJ, Dwyer S, Gormley P, et al De novo mutations in schizophrenia implicate synaptic networks. Nature. 2014;506(7487):179–84. 10.1038/nature12929 24463507 PMC4237002

[78] Toma C, Shaw AD, Allcock RJN, Heath A, Pierce KD, Mitchell PB, et al An examination of multiple classes of rare variants in extended families with bipolar disorder. Translational psychiatry. 2018;8(1):65 10.1038/s41398-018-0113-y . 29531218 PMC5847564

[79] Huang N, Lee I, Marcotte EM, Hurles ME. Characterising and predicting haploinsufficiency in the human genome. PLoS genetics. 2010;6(10):e1001154 10.1371/journal.pgen.1001154 20976243 PMC2954820

[80] Ross LA, Del Bene VA, Molholm S, Jae Woo Y, Andrade GN, Abrahams BS, et al Common variation in the autism risk gene CNTNAP2, brain structural connectivity and multisensory speech integration. Brain and language. 2017;174:50–60. 10.1016/j.bandl.2017.07.005 . 28738218

[81] Toma C, Hervas A, Torrico B, Balmana N, Salgado M, Maristany M, et al Analysis of two language-related genes in autism: a case-control association study of FOXP2 and CNTNAP2. Psychiatric genetics. 2013;23(2):82–5. 10.1097/YPG.0b013e32835d6fc6 . 23277129

[82] Werling AM, Bobrowski E, Taurines R, Gundelfinger R, Romanos M, Grunblatt E, et al CNTNAP2 gene in high functioning autism: no association according to family and meta-analysis approaches. J Neural Transm (Vienna). 2016;123(3):353–63. 10.1007/s00702-015-1458-5 . 26559825

[83] Torrico B, Chiocchetti AG, Bacchelli E, Trabetti E, Hervas A, Franke B, et al Lack of replication of previous autism spectrum disorder GWAS hits in European populations. Autism Res. 2017;10(2):202–11. 10.1002/aur.1662 . 27417655

[84] Murdoch JD, Gupta AR, Sanders SJ, Walker MF, Keaney J, Fernandez TV, et al No evidence for association of autism with rare heterozygous point mutations in Contactin-Associated Protein-Like 2 (CNTNAP2), or in Other Contactin-Associated Proteins or Contactins. PLoS genetics. 2015;11(1):e1004852 10.1371/journal.pgen.1004852 25621974 PMC4306541

[85] Canali G, Garcia M, Hivert B, Pinatel D, Goullancourt A, Oguievetskaia K, et al Genetic variants in autism-related CNTNAP2 impair axonal growth of cortical neurons. Human molecular genetics. 2018;27(11):1941–54. 10.1093/hmg/ddy102 . 29788201

[86] de Leeuw CA, Mooij JM, Heskes T, Posthuma D. MAGMA: generalized gene-set analysis of GWAS data. PLoS computational biology. 2015;11(4):e1004219 10.1371/journal.pcbi.1004219 25885710 PMC4401657

[87] Demontis D, Walters RK, Martin J, Mattheisen M, Als TD, Agerbo E, et al Discovery Of The First Genome-Wide Significant Risk Loci For ADHD. bioRxiv. 2017 10.1101/145581 PMC6481311 30478444

[88] Autism Spectrum Disorders Working Group of The Psychiatric Genomics C. Meta-analysis of GWAS of over 16,000 individuals with autism spectrum disorder highlights a novel locus at 10q24.32 and a significant overlap with schizophrenia. Molecular autism. 2017;8:21 10.1186/s13229-017-0137-9 28540026 PMC5441062

[89] Major Depressive Disorder Working Group of the Psychiatric GC, Ripke S, Wray NR, Lewis CM, Hamilton SP, Weissman MM, et al A mega-analysis of genome-wide association studies for major depressive disorder. Molecular psychiatry. 2013;18(4):497–511. 10.1038/mp.2012.21 22472876 PMC3837431

[90] Schizophrenia Working Group of the Psychiatric Genomics C. Biological insights from 108 schizophrenia-associated genetic loci. Nature. 2014;511(7510):421–7. 10.1038/nature13595 25056061 PMC4112379

[91] Stahl E, Forstner A, McQuillin A, Ripke S, Ophoff R, Scott L, et al Genomewide association study identifies 30 loci associated with bipolar disorder. bioRxiv. 2017 10.1101/173062

[92] International Obsessive Compulsive Disorder Foundation Genetics C, Studies OCDCGA. Revealing the complex genetic architecture of obsessive-compulsive disorder using meta-analysis. Molecular psychiatry. 2018;23(5):1181–8. 10.1038/mp.2017.154 . 28761083 PMC6660151

[93] Duncan L, Yilmaz Z, Gaspar H, Walters R, Goldstein J, Anttila V, et al Significant Locus and Metabolic Genetic Correlations Revealed in Genome-Wide Association Study of Anorexia Nervosa. The American journal of psychiatry. 2017;174(9):850–8. 10.1176/appi.ajp.2017.16121402 28494655 PMC5581217

[94] Purcell S, Neale B, Todd-Brown K, Thomas L, Ferreira MA, Bender D, et al PLINK: a tool set for whole-genome association and population-based linkage analyses. American journal of human genetics. 2007;81(3):559–75. 10.1086/519795 17701901 PMC1950838

[95] Dopazo J, Amadoz A, Bleda M, Garcia-Alonso L, Aleman A, Garcia-Garcia F, et al 267 Spanish Exomes Reveal Population-Specific Differences in Disease-Related Genetic Variation. Molecular biology and evolution. 2016;33(5):1205–18. 10.1093/molbev/msw005 26764160 PMC4839216

[96] Puente XS, Bea S, Valdes-Mas R, Villamor N, Gutierrez-Abril J, Martin-Subero JI, et al Non-coding recurrent mutations in chronic lymphocytic leukaemia. Nature. 2015;526(7574):519–24. 10.1038/nature14666 . 26200345

[97] Toma C, Torrico B, Hervas A, Valdes-Mas R, Tristan-Noguero A, Padillo V, et al Exome sequencing in multiplex autism families suggests a major role for heterozygous truncating mutations. Molecular psychiatry. 2014;19(7):784–90. 10.1038/mp.2013.106 . 23999528

[98] Li J, Cai T, Jiang Y, Chen H, He X, Chen C, et al Genes with de novo mutations are shared by four neuropsychiatric disorders discovered from NPdenovo database. Molecular psychiatry. 2016;21(2):290–7. 10.1038/mp.2015.40 25849321 PMC4837654

[99] Turner TN, Yi Q, Krumm N, Huddleston J, Hoekzema K, HA FS, et al denovo-db: a compendium of human de novo variants. Nucleic acids research. 2017;45(D1):D804–D11. 10.1093/nar/gkw865 27907889 PMC5210614

[100] Adams LJ, Mitchell PB, Fielder SL, Rosso A, Donald JA, Schofield PR. A susceptibility locus for bipolar affective disorder on chromosome 4q35. Am J Hum Genet. 1998;62(5):1084–91. 10.1086/301826 . 9545396 PMC1377083

[101] Badenhop RF, Moses MJ, Scimone A, Adams LJ, Kwok JB, Jones AM, et al Genetic refinement and physical mapping of a 2.3 Mb probable disease region associated with a bipolar affective disorder susceptibility locus on chromosome 4q35. Am J Med Genet B Neuropsychiatr Genet. 2003;117(1):23–32. 10.1002/ajmg.b.10023 . 12555231

[102] Fullerton JM, Donald JA, Mitchell PB, Schofield PR. Two-Dimensional Genome Scan Identifies Multiple Genetic Interactions in Bipolar Affective Disorder. Biol Psychiatry. 2010;67(5):478–86. 10.1016/j.biopsych.2009.10.022 . 20022591

[103] Fullerton JM, Liu Z, Badenhop RF, Scimone A, Blair IP, Van Herten M, et al Genome screen of 15 Australian bipolar affective disorder pedigrees supports previously identified loci for bipolar susceptibility genes. Psychiatr Genet. 2008;18(4):156–61. 10.1097/YPG.0b013e3282fa1861 . 18628676

[104] McAuley EZ, Blair IP, Liu Z, Fullerton JM, Scimone A, Van Herten M, et al A genome screen of 35 bipolar affective disorder pedigrees provides significant evidence for a susceptibility locus on chromosome 15q25-26. Mol Psychiatry. 2009;14(5):492–500. 10.1038/sj.mp.4002146 . 18227837

[105] National Institute of Mental Health. NIMH Genetics Initiative: Family Interview for Genetic Studies (FIGS). Rockville, MD1992.

[106] Nurnberger JI Jr., Blehar MC, Kaufmann CA, York-Cooler C, Simpson SG, Harkavy-Friedman J, et al Diagnostic interview for genetic studies. Rationale, unique features, and training. NIMH Genetics Initiative. Archives of general psychiatry. 1994;51(11):849–59; discussion 63–4. . 7944874 10.1001/archpsyc.1994.03950110009002

