# Supplementary material for: Comprehensive cross-disorder analyses of CNTNAP2 suggest it is unlikely to be a primary risk gene for psychiatric disorders
Source: PLoS Genet. 2018 Dec 26;14(12):e1007535. doi: 10.1371/journal.pgen.1007535 (PMC6324819; doi:10.1371/journal.pgen.1007535)
Supplement: S1 Fig — (DOCX) [file pgen.1007535.s001.docx]

**S1 Figure. Association plots of *CNTNAP2* in each of the seven psychiatric disorders examined using GWAS summary statistics of the PGC data sets.** The y-axis indicates the significance of association as the negative logarithm of the p-value (–log *P-Value*), and x-axis indicates the physical position along the gene in megabases (Mb). The name of the most significant SNP is indicated in each plot. Linkage disequilibrium between SNPs is calculated using the 1,000 genomes European population.

**
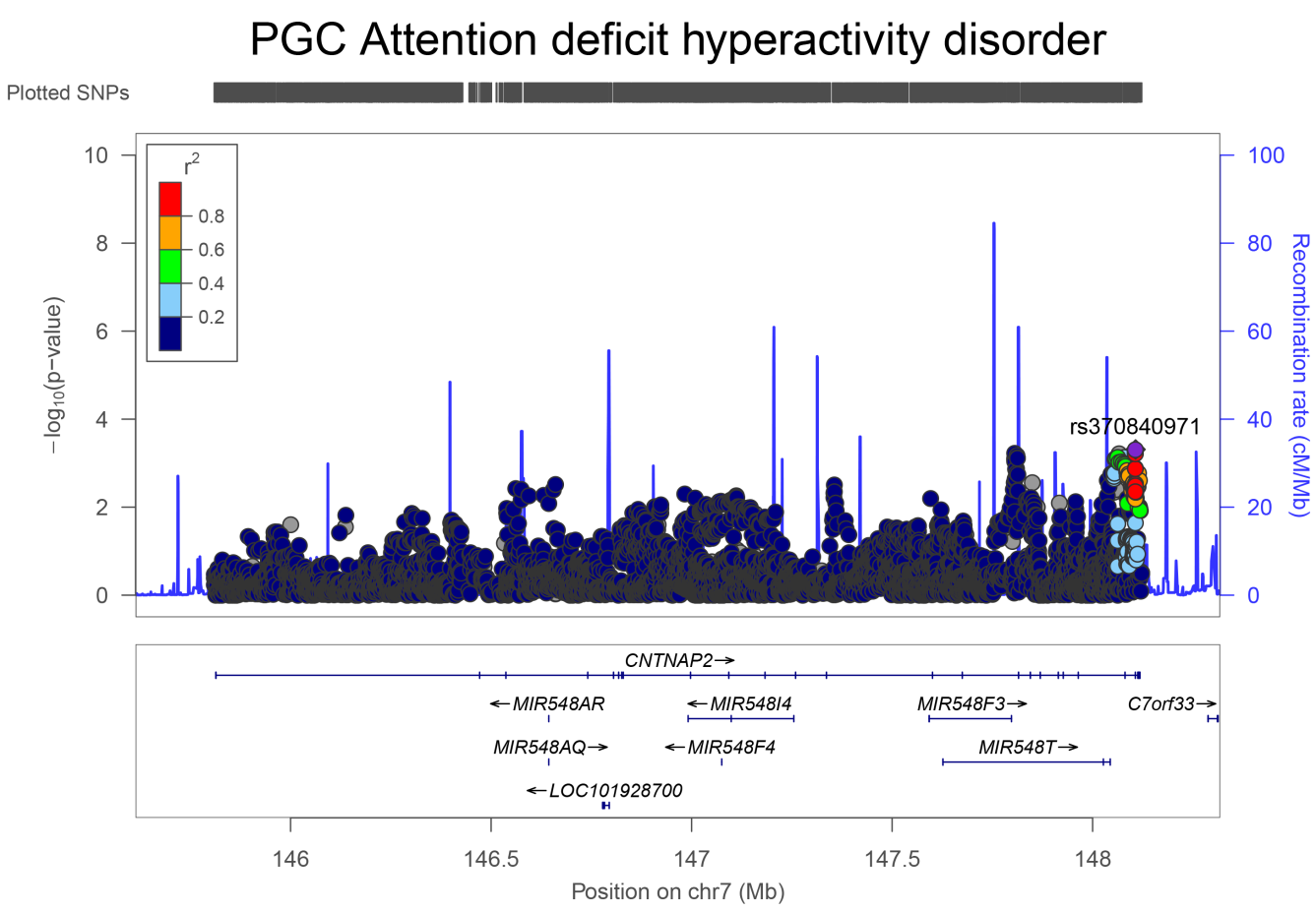
**

**
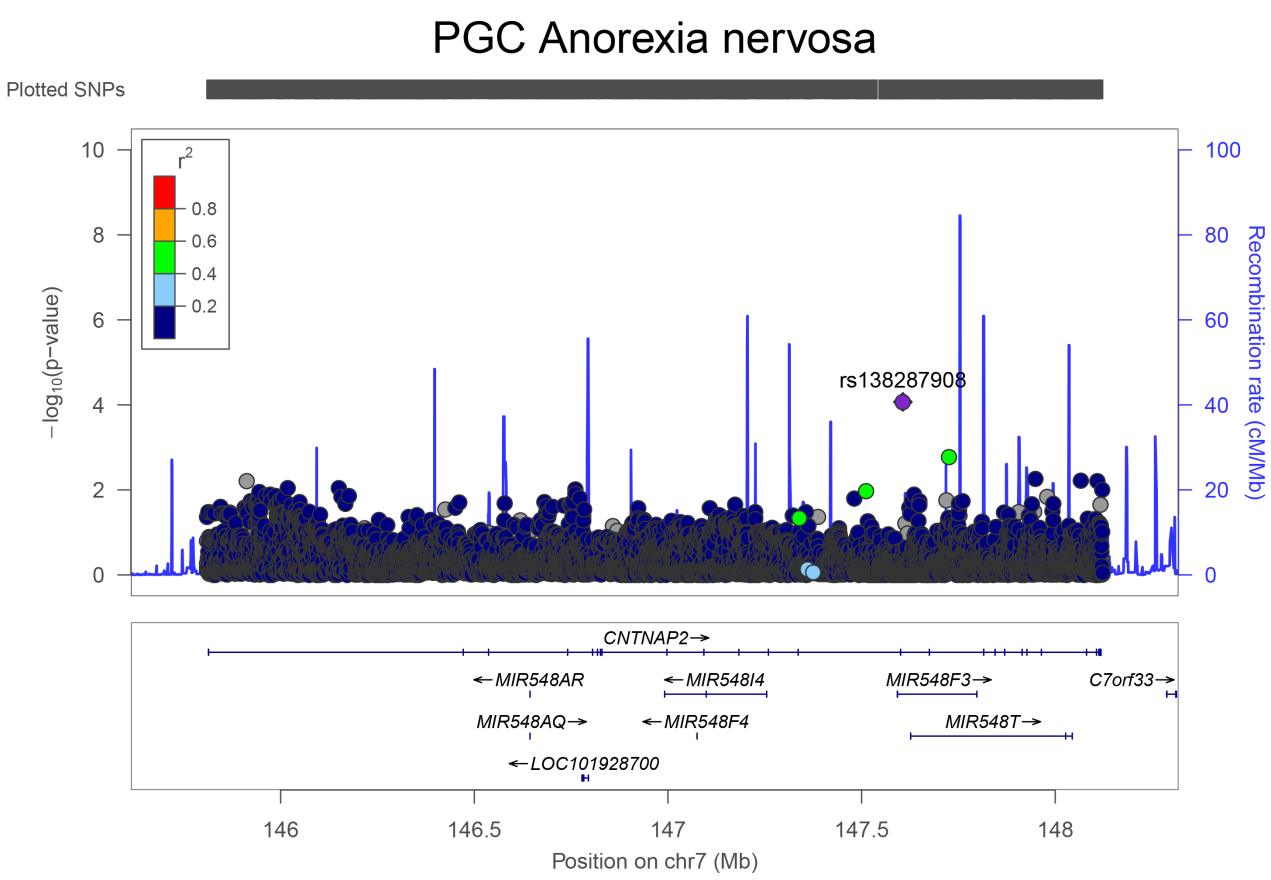
**

**
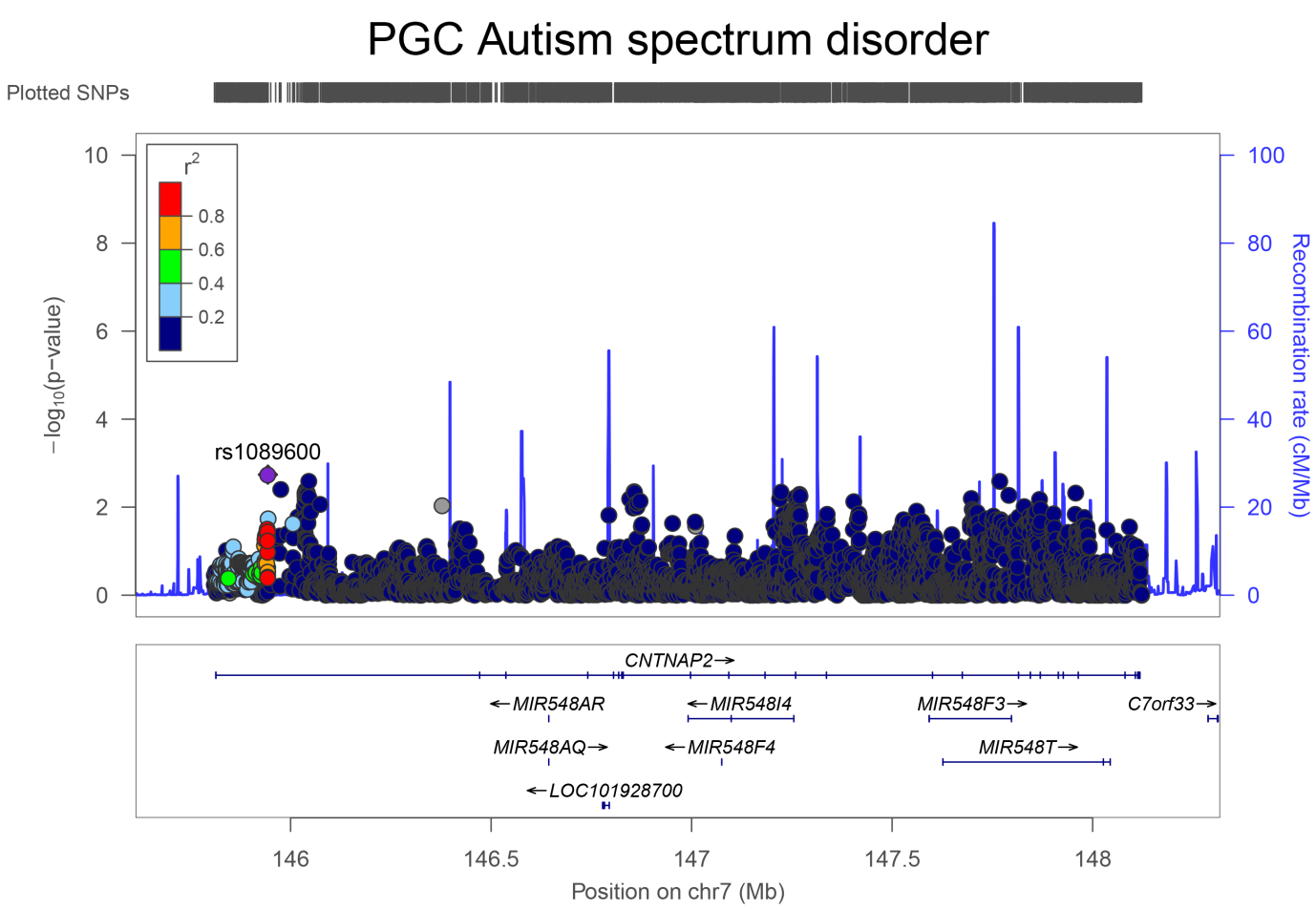
**

**
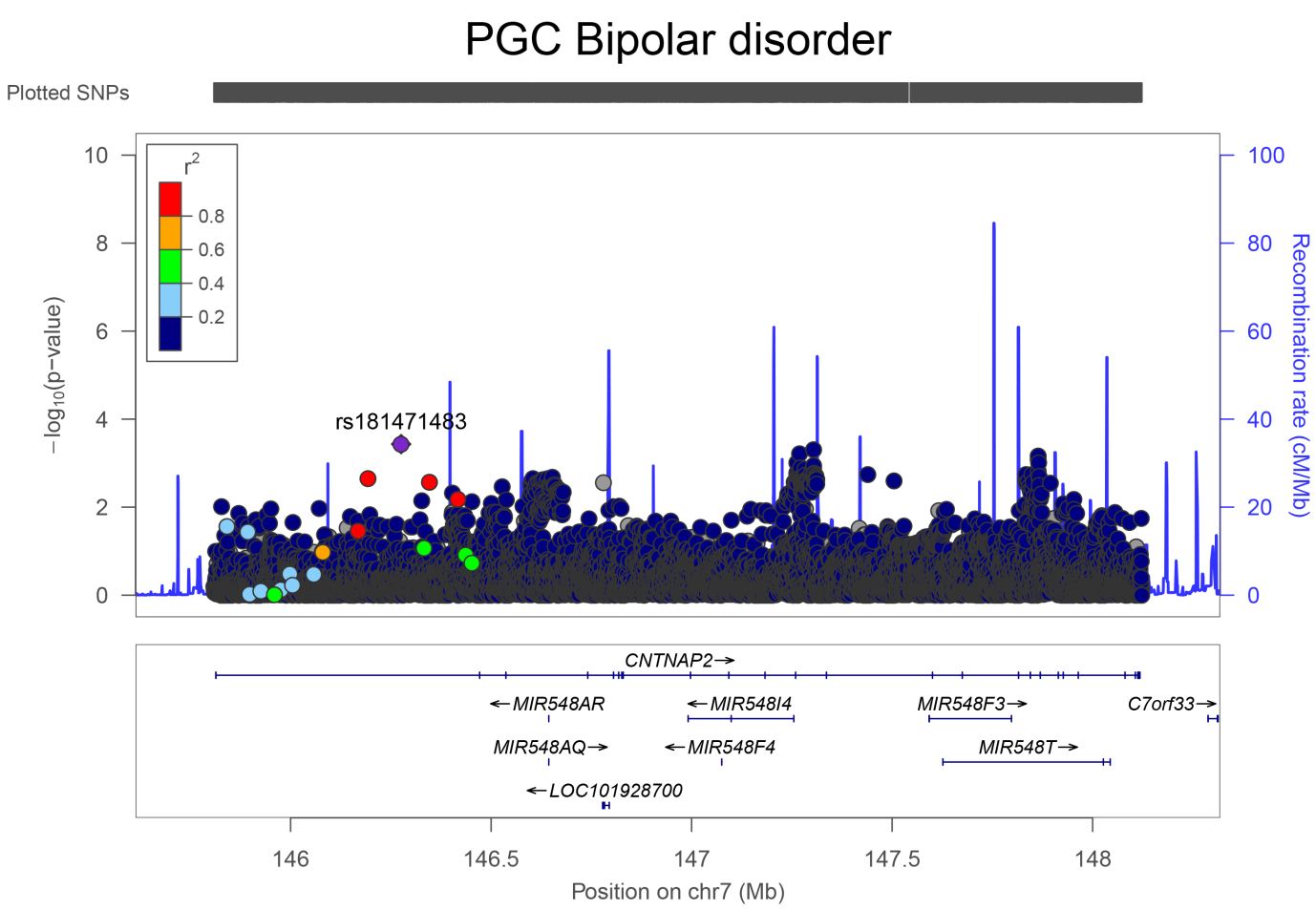
**

**
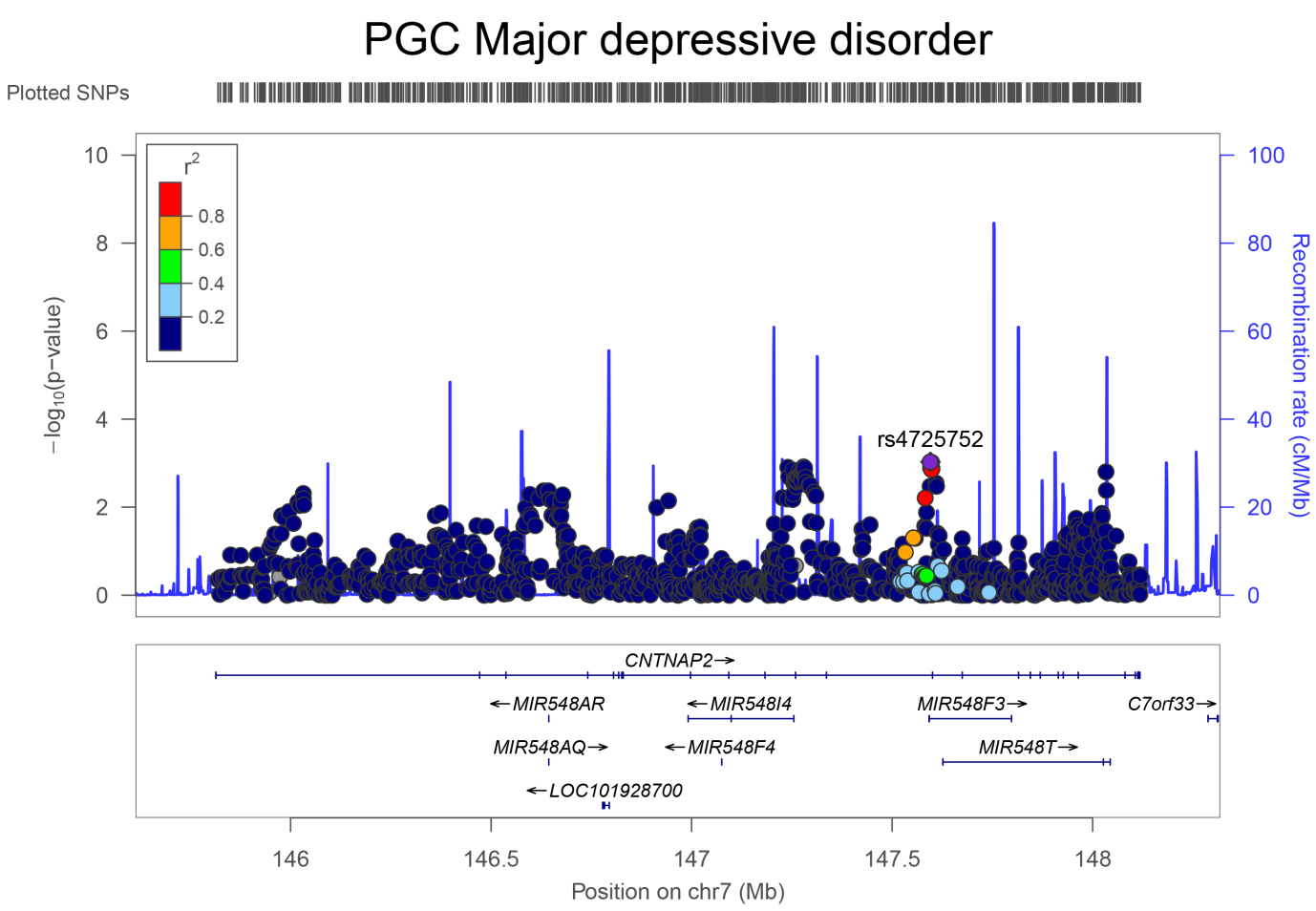
**

**
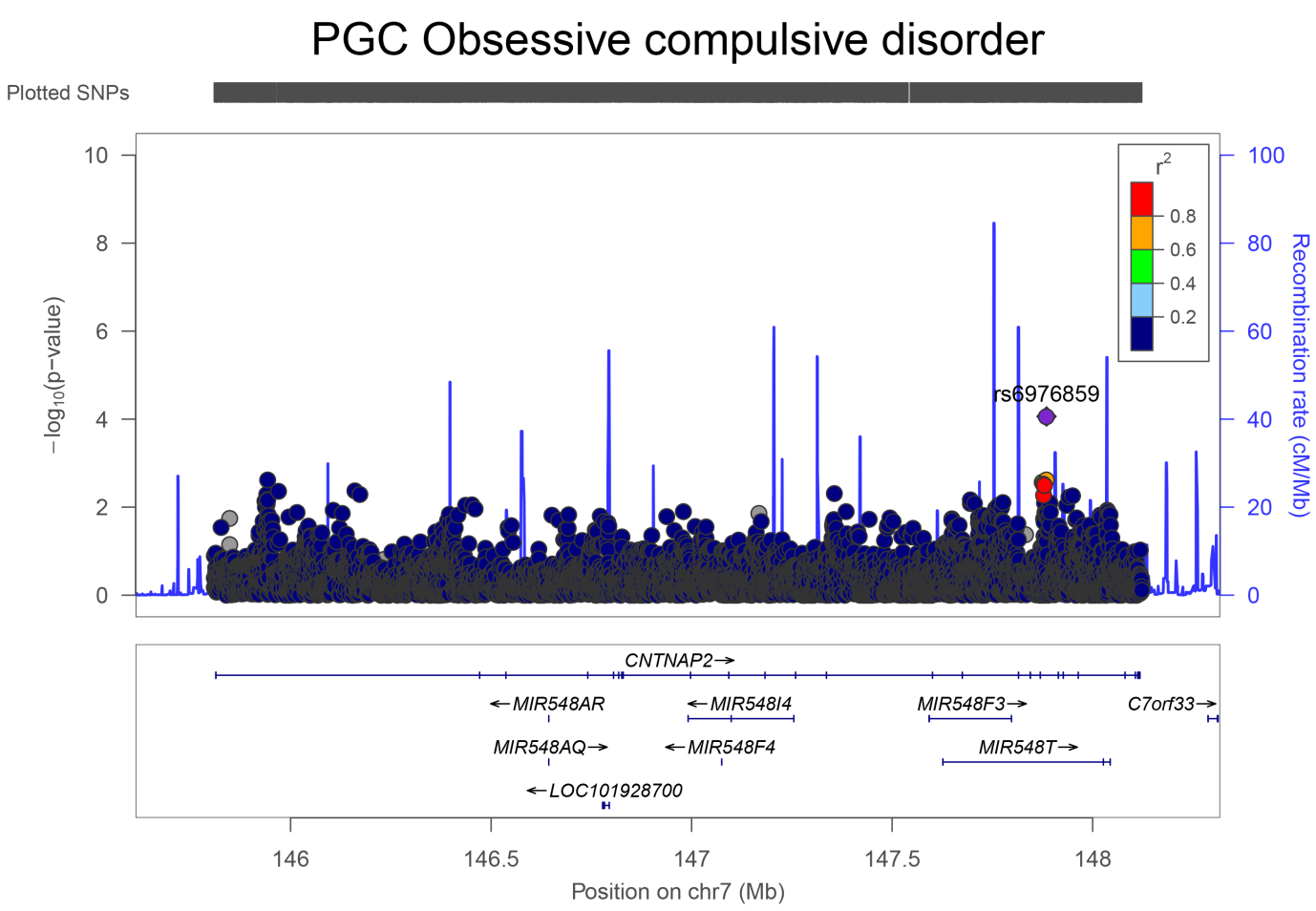
**

**
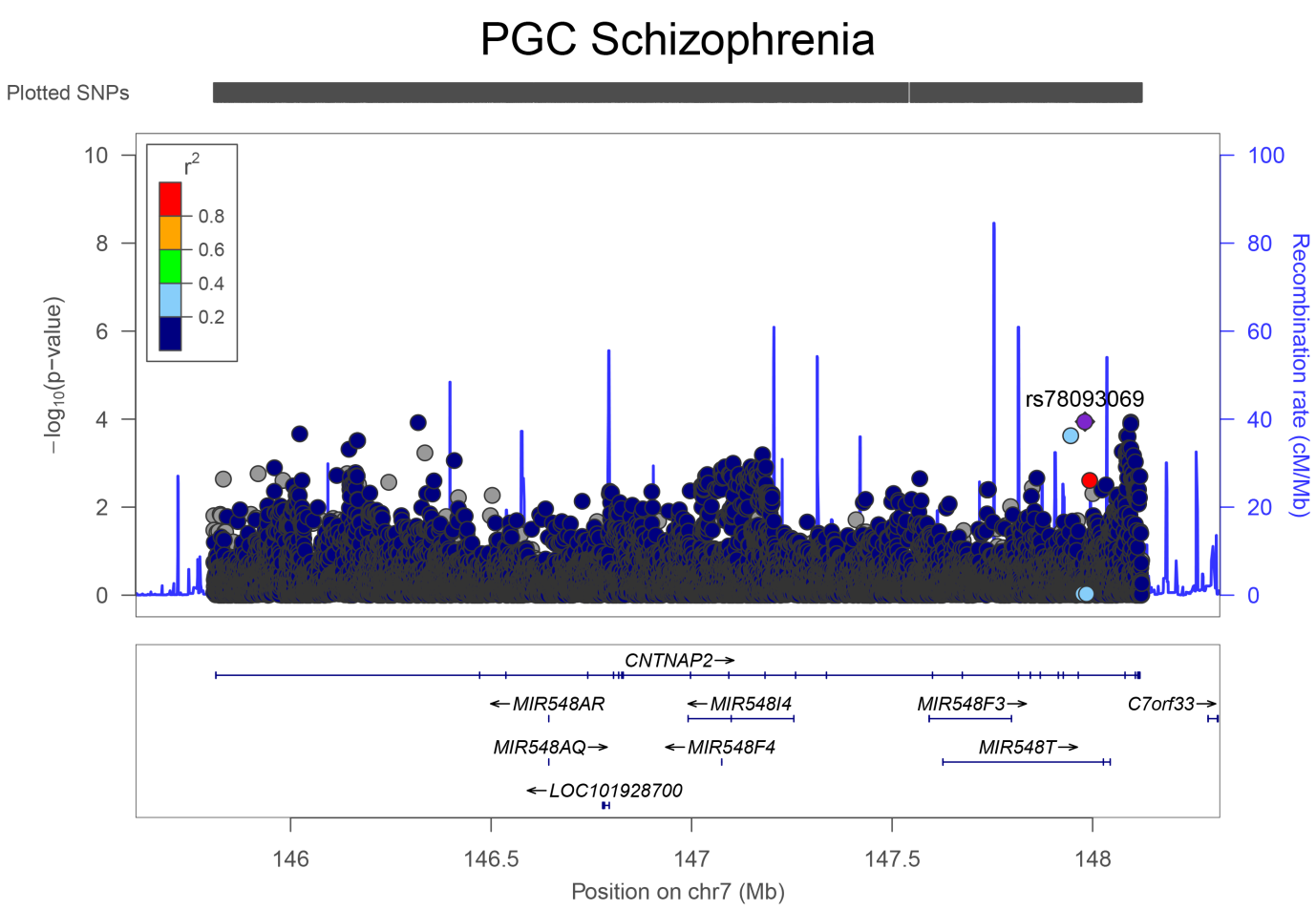
**
