# Supplementary material for: Comprehensive cross-disorder analyses of CNTNAP2 suggest it is unlikely to be a primary risk gene for psychiatric disorders
Source: PLoS Genet. 2018 Dec 26;14(12):e1007535. doi: 10.1371/journal.pgen.1007535 (PMC6324819; doi:10.1371/journal.pgen.1007535)
Supplement: S2 Fig — (DOCX) [file pgen.1007535.s002.docx]

**S2 Figure. Cross-disorder association plot of *CNTNAP2* common variants predicted to be functional (63 SNPs).** The y-axis indicates the significance of association as the negative logarithm of the p-value (–log *P-Value*), and x-axis indicates the physical position along the gene in megabases (Mb). Linkage disequilibrium between SNPs is calculated using the 1,000 genomes European population. *P-values* are derived from meta-analysis results across seven psychiatric disorders, which are presented numerically in Table 4. The position of the most significant SNP (rs4725756) is shown.

**
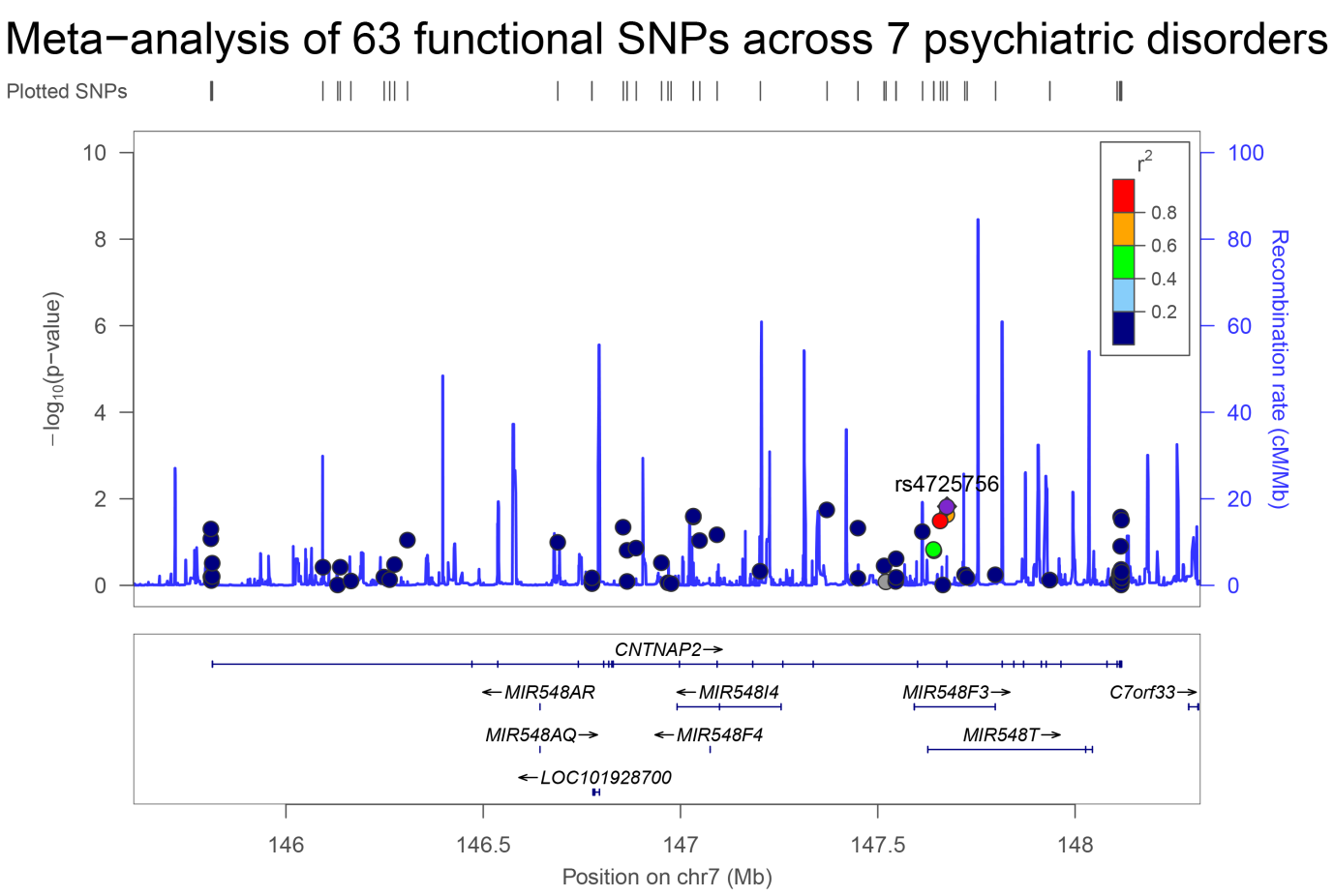
**
