# Supplementary material for: Comprehensive cross-disorder analyses of CNTNAP2 suggest it is unlikely to be a primary risk gene for psychiatric disorders
Source: PLoS Genet. 2018 Dec 26;14(12):e1007535. doi: 10.1371/journal.pgen.1007535 (PMC6324819; doi:10.1371/journal.pgen.1007535)
Supplement: S1 Table — (DOCX) [file pgen.1007535.s003.docx]

**S1 Table. Gene-based analysis of predicted functional SNPs across seven psychiatric disorders.** P-values were calculated using MAGMA and Bonferroni corrected for multiple testing. A cross-disorder meta-analysis was also performed across the seven psychiatric disorders.

| **Disease** | **N SNPs** | **Zstat** | ***P-value*** | **Corrected *P-value*** |
| --- | --- | --- | --- | --- |
| ADHD^a^ | 51 | 2.597 | 0.0047 | ***0.0329*** |
| AN^a^ | 57 | -0.85 | 0.8 | 1 |
| ASD^a^ | 40 | 0.189 | 0.42 | 1 |
| BD^a^ | 60 | -1.129 | 0.87 | 1 |
| MDD^b^ | 22 | -1.163 | 0.87 | 1 |
| OCD^a^ | 57 | 0.028 | 0.48 | 1 |
| SCZ^a^ | 60 | 1.7 | 0.044 | 0.3 |
| Cross-disorder | | 1.18 | 0.11 |  |

Abbreviations: ADHD, attention-deficit/hyperactivity disorder; AN, anorexia nervosa; ASD, Autism spectrum disorder; BD, bipolar disorder; MDD, major depressive disorder; OCD, obsessive compulsive disorder; SCZ, schizophrenia; ^a^, European individuals from the PGC2 data sets; ^b^, European individuals from the PGC1 data sets.
