# Supplementary material for: Comprehensive cross-disorder analyses of CNTNAP2 suggest it is unlikely to be a primary risk gene for psychiatric disorders
Source: PLoS Genet. 2018 Dec 26;14(12):e1007535. doi: 10.1371/journal.pgen.1007535 (PMC6324819; doi:10.1371/journal.pgen.1007535)
Supplement: S2 Table — (DOCX) [file pgen.1007535.s004.docx]

**S2 Table. Full list of d*e novo* variants in *CNTNAP2* gene.** Identified via different sequencing projects in psychiatric disorders (http://www.wzgenomics.cn/NPdenovo and http://denovo-db.gs.washington.edu/denovo-db), which comprise data for the following sample: autism spectrum disorder (6,171 families), schizophrenia: (1,164 families), epilepsy (647 families), intellectual disability (1,101 families), developmental disorders (4,293 families) and controls (2,163). Genomic positions at chromosome 7 are indicated according to the GRCh37/hg19 assembly of the UCSC Genome Browser (genome.ucsc.edu).

| **Database** | **Sample** | **Method** | **Phenotype** | **Position** | **Variant** | **Type/Function** | **CADD** | **dbSNP ID** |
| --- | --- | --- | --- | --- | --- | --- | --- | --- |
| denovo-db | DDD4K.00451 | WES | DD | 145813992 | C>A | Synonymous/p.(G8=) | 11.011 | - |
| denovo-db | DDD4K.01230 | WES | DD | 145813992 | C>A | Synonymous/p.(G8=) | 11.011 | - |
| denovo-db | gonl-98c | WGS | control | 145897669 | T>C | Intron/NA | 1.176 | - |
| denovo-db | 2-0256-004 | WGS | ASD | 145903449 | C>T | Intron/NA | 11.471 | - |
| denovo-db | 1-0484-003 | WGS | ASD | 145915359 | T>C | Intron/NA | 2.833 | - |
| denovo-db | 2-1477-003 | WGS | ASD | 145938253 | A>G | Intron/NA | 8.919 | - |
| denovo-db | gonl-86c | WGS | control | 145947556 | G>A | Intron/NA | 4.6 | - |
| denovo-db | AU015903 | WGS | ASD | 145952674 | T>C | Intron/NA | 2.05 | - |
| Npdenovo | [1-0366_003](http://www.wzgenomics.cn/NPdenovo/clinical_info.php?PubMed_ID=25621899&proband_ID=1-0366_003) | WGS | ASD | 145970049 | C>A | Intron/NA |  |  |
| denovo-db | 1-0756-005 | WGS | ASD | 146007616 | T>C | Intron/NA | 2.319 | - |
| denovo-db | AU057503 | WGS | ASD | 146042001 | C>A | Intron/NA | 2.452 | - |
| denovo-db | 2-0286-003 | WGS | ASD | 146085273 | C>T | Intron/NA | 0.454 | - |
| denovo-db | 1-0604-003 | WGS | ASD | 146091132 | C>T | Intron/NA | 4.021 | - |
| denovo-db | 2-0109-003 | WGS | ASD | 146114411 | A>G | Intron/NA | 19.97 | - |
| denovo-db | 1-0701-003 | WGS | ASD | 146162826 | C>T | Intron/NA | 0.394 | - |
| denovo-db | AU058105 | WGS | ASD | 146208335 | T>G | Intron/NA | 1.099 | - |
| denovo-db | 2-1644-004 | WGS | ASD | 146267480 | G>A | Intron/NA | 2.554 | - |
| denovo-db | AU026412 | WGS | ASD | 146290577 | A>G | Intron/NA | 1.816 | - |
| Npdenovo | [A2](http://www.wzgenomics.cn/NPdenovo/clinical_info.php?PubMed_ID=-&proband_ID=A2) | WGS | ASD | 146295830 | T>C | Intron/NA |  |  |
| denovo-db | 5-0074-003 | WGS | ASD | 146301421 | A>G | Intron/NA | 1.935 | - |
| denovo-db | 7-0167-003 | WGS | ASD | 146304967 | C>A | Intron/NA | 0.473 | - |
| denovo-db | 1-0652-003 | WGS | ASD | 146354396 | A>G | Intron/NA | 8.002 | - |
| denovo-db | 7-0102-003 | WGS | ASD | 146359965 | C>T | Intron/NA | 1.15 | - |
| denovo-db | AU057503 | WGS | ASD | 146370640 | G>C | Intron/NA | 0.565 | - |
| denovo-db | AU066206 | WGS | ASD | 146371397 | C>T | Intron/NA | 0.768 | - |
| denovo-db | AU3692301 | WGS | ASD | 146383772 | T>C | Intron/NA | 5.765 | - |
| denovo-db | 1-0032-003 | WGS | ASD | 146388346 | T>C | Intron/NA | 5.566 | - |
| denovo-db | AU4306302 | WGS | ASD | 146398475 | A>G | Intron/NA | 0.824 | - |
| denovo-db | AU4239301 | WGS | ASD | 146481913 | C>T | Intron/NA | 1.364 | - |
| denovo-db | AU4231301 | WGS | ASD | 146501606 | A>G | Intron/NA | 0.02 | - |
| denovo-db | 1-0564-003 | WGS | ASD | 146524105 | A>G | Intron/NA | 4.586 | - |
| Npdenovo | [1-0366_003](http://www.wzgenomics.cn/NPdenovo/clinical_info.php?PubMed_ID=25621899&proband_ID=1-0366_003) | WGS | ASD | 146531798 | C>A | Intron/NA |  |  |
| denovo-db | 2-0149-004 | WGS | ASD | 146536632 | T>G | Intron/NA | 21.701 | - |
| denovo-db | 1-0556-003 | WGS | ASD | 146556079 | C>T | Intron/NA | 4.911 | - |
| denovo-db | AU3889305 | WGS | ASD | 146577028 | T>G | Intron/NA | 1.407 | [rs19289794](http://www.ncbi.nlm.nih.gov/projects/SNP/snp_ref.cgi?searchType=adhoc_search&type=rs&rs=rs192897940)0 |
| denovo-db | gonl-197c | WGS | control | 146581057 | A>G | Intron/NA | 12.641 | - |
| denovo-db | AU2072302 | WGS | ASD | 146595063 | T>C | Intron/NA | 6.56 | - |
| denovo-db | AU4356302 | WGS | ASD | 146656150 | C>T | Intron/NA | 0.339 | - |
| denovo-db | 1-0841-003 | WGS | ASD | 146667283 | A>G | Intron/NA | 0.338 | - |
| denovo-db | 1-0487-003 | WGS | ASD | 146720427 | T>C | Intron/NA | 1.06 | - |
| denovo-db | gonl-207c | WGS | control | 146739179 | G>A | Intron/NA | 2.567 | - |
| denovo-db | 2-0129-004 | WGS | ASD | 146786651 | G>C | Intron/NA | 12.16 | - |
| denovo-db | 1-0874-003 | WGS | ASD | 146791618 | C>T | Intron/NA | 1.232 | - |
| denovo-db | AU3782303 | WGS | ASD | 146796007 | C>A | Intron/NA | 1.422 | - |
| denovo-db | 1-0465-003 | WGS | ASD | 146796293 | A>G | Intron/NA | 2.145 | - |
| denovo-db | 2-0278-003 | WGS | ASD | 146796410 | T>C | Intron/NA | 0.704 | - |
| Npdenovo | [14304](http://www.wzgenomics.cn/NPdenovo/clinical_info.php?PubMed_ID=25363768&proband_ID=14304) | WES | control | 146805224 | ->G | Intron/NA |  |  |
| denovo-db | 14304.s1 | WES | control | 146805225 | T>TG | Intron/NA | -1 | - |
| Npdenovo | [2-0081_004](http://www.wzgenomics.cn/NPdenovo/clinical_info.php?PubMed_ID=25621899&proband_ID=2-0081_004) | WGS | ASD | 146819343 | A>G | Intron/NA |  |  |
| denovo-db | 1-0046-003 | WGS | ASD | 146833088 | G>A | Intron/NA | 2.277 | - |
| denovo-db | AU011021 | WGS | ASD | 146834880 | A>G | Intron/NA | 3.363 | - |
| denovo-db | AU4435301 | WGS | ASD | 146888860 | A>T | Intron/NA | 8.03 | - |
| Npdenovo | [1-0279_003](http://www.wzgenomics.cn/NPdenovo/clinical_info.php?PubMed_ID=25621899&proband_ID=1-0279_003) | WGS | ASD | 146892609 | ->AATTTATAG  ATTTGGGCAGAA | Intron/NA |  |  |
| denovo-db | 2-1629-003 | WGS | ASD | 146900950 | A>T | Intron/NA | 6.41 | - |
| denovo-db | 7-0100-004 | WGS | ASD | 146932119 | G>A | Intron/NA | 3.658 | - |
| denovo-db | 2-1507-003 | WGS | ASD | 146943173 | T>C | Intron/NA | 0.35 | - |
| denovo-db | 1-0756-005 | WGS | ASD | 146954511 | G>C | Intron/NA | 1.667 | - |
| denovo-db | 3-0437-000 | WGS | ASD | 146984568 | C>A | Intron/NA | 1.247 | - |
| denovo-db | 2-0256-004 | WGS | ASD | 146994781 | T>C | Intron/NA | 2.253 | - |
| denovo-db | AU3787302 | WGS | ASD | 147026531 | T>A | Intron/NA | 3.661 | - |
| denovo-db | 2-0057-003 | WGS | ASD | 147070547 | T>C | Intron/NA | 5.14 | - |
| denovo-db | 11002.p1 | WGS | ASD | 147113436 | A>G | Intron/NA | 5.857 | - |
| denovo-db | 2-1222-003 | WGS | ASD | 147122126 | C>T | Intron/NA | 5.011 | - |
| denovo-db | 1-0744-003 | WGS | ASD | 147134348 | ATGT>AT | Intron/NA | -1 | - |
| denovo-db | 3-0456-000 | WGS | ASD | 147138133 | T>C | Intron/NA | 1.739 | - |
| denovo-db | gonl-83c | WGS | control | 147157205 | C>T | Intron/NA | 1.158 | - |
| denovo-db | 2-0323-003 | WGS | ASD | 147165457 | ATTAC>A | Intron/NA | -1 | - |
| denovo-db | gonl-52c | WGS | control | 147170174 | C>T | Intron/NA | 2.679 | - |
| denovo-db | 13637.p1 | WGS | ASD | 147188561 | A>C | Intron/NA | 4.943 | - |
| denovo-db | AU3506303 | WGS | ASD | 147220799 | C>A | Intron/NA | 0.29 | - |
| denovo-db | gonl-77c | WGS | control | 147225572 | G>A | Intron/NA | 1.759 | - |
| denovo-db | 1-0261-004 | WGS | ASD | 147251690 | A>T | Intron/NA | 3.402 | - |
| denovo-db | 2-1605-003 | WGS | ASD | 147321475 | T>TTATA | Intron/NA | -1 | - |
| denovo-db | 2-1337-003 | WGS | ASD | 147324378 | C>T | Intron/NA | 8.179 | - |
| denovo-db | 2-0273-003 | WGS | ASD | 147329307 | AT>A | Intron/NA | -1 | - |
| denovo-db | 1-0582-003 | WGS | ASD | 147334624 | CTGT>CT | Intron/NA | -1 | - |
| denovo-db | 1-0272-004 | WGS | ASD | 147340738 | G>A | Intron/NA | 19.65 | - |
| denovo-db | AU1635302 | WGS | ASD | 147347156 | C>T | Intron/NA | 2.645 | - |
| denovo-db | AU3122301 | WGS | ASD | 147388092 | T>C | Intron/NA | 3.928 | - |
| denovo-db | 1-0006-004 | WGS | ASD | 147402907 | C>A | Intron/NA | 0.52 | - |
| denovo-db | 1-0473-003 | WGS | ASD | 147413074 | CT>C | Intron/NA | -1 | - |
| denovo-db | AU3730301 | WGS | ASD | 147431366 | C>T | Intron/NA | 0.145 | - |
| denovo-db | 2-1299-003 | WGS | ASD | 147439038 | C>A | Intron/NA | 2.904 | - |
| Npdenovo | [1-0366_003](http://www.wzgenomics.cn/NPdenovo/clinical_info.php?PubMed_ID=25621899&proband_ID=1-0366_003) | WGS | ASD | 147442748 | A>G | Intron/NA |  |  |
| denovo-db | gonl-33c | WGS | control | 147454954 | C>T | Intron/NA | 0.12 | - |
| denovo-db | 2-1525-003 | WGS | ASD | 147467228 | T>C | Intron/NA | 1.257 | - |
| denovo-db | gonl-95c | WGS | control | 147489560 | A>G | Intron/NA | 1.525 | - |
| denovo-db | 1-0368-004 | WGS | ASD | 147490259 | C>T | Intron/NA | 0.461 | - |
| denovo-db | 7-0273-003 | WGS | ASD | 147493754 | A>G | Intron/NA | 11.511 | - |
| denovo-db | gonl-207c | WGS | control | 147515748 | T>C | Intron/NA | 5.104 | - |
| denovo-db | 12449.s1 | WGS | control | 147555745 | C>T | Intron/NA | 3.326 | - |
| denovo-db | 1-0591-003 | WGS | ASD | 147564863 | G>T | Intron/NA | 1.354 | - |
| denovo-db | 13069.p1 | WGS | ASD | 147595528 | C>G | Intron/NA | 7.687 | - |
| denovo-db | 3-0065-000 | WGS | ASD | 147650633 | A>C | Intron/NA | 3.043 | - |
| denovo-db | 1-0627-006 | WGS | ASD | 147662660 | G>A | Intron/NA | 7.527 | [rs191006031](http://www.ncbi.nlm.nih.gov/projects/SNP/snp_ref.cgi?searchType=adhoc_search&type=rs&rs=rs191006031) |
| denovo-db | 2-1577-003 | WGS | ASD | 147664927 | C>A | Intron/NA | 6.315 | - |
| denovo-db | 13185.p1 | WGS | ASD | 147668572 | C>T | Intron/NA | 3.266 | - |
| denovo-db | 1-0559-004 | WGS | ASD | 147674357 | A>G | Intron/NA | 3.771 | - |
| denovo-db | 1-0232-004 | WGS | ASD | 147694858 | T>C | Intron/NA | 2.646 | [rs143805310](http://www.ncbi.nlm.nih.gov/projects/SNP/snp_ref.cgi?searchType=adhoc_search&type=rs&rs=rs143805310) |
| denovo-db | 3-0446-000 | WGS | ASD | 147701849 | C>T | Intron/NA | 10.561 | - |
| denovo-db | 1-0218-003 | WGS | ASD | 147703937 | C>T | Intron/NA | 1.762 | - |
| denovo-db | 2-0297-003 | WGS | ASD | 147715897 | T>A | Intron/NA | 9.565 | - |
| denovo-db | AU2975302 | WGS | ASD | 147750770 | T>C | Intron/NA | 0.036 | - |
| denovo-db | AU3875301 | WGS | ASD | 147757888 | G>A | Intron/NA | 0.727 | - |
| denovo-db | 5-0117-003 | WGS | ASD | 147759105 | GA>GATGATA | Intron/NA | -1 | - |
| denovo-db | AU3052301 | WGS | ASD | 147760036 | GAAATGAAAATG  >GAAATG | Intron/NA | -1 | - |
| denovo-db | 5-0046-003 | WGS | ASD | 147775022 | T>C | Intron/NA | 10.341 | - |
| denovo-db | 1-0113-003 | WGS | ASD | 147782673 | G>A | Intron/NA | 0.419 | - |
| denovo-db | 7-0161-003 | WGS | ASD | 147794074 | G>A | Intron/NA | 3.783 | - |
| denovo-db | 3-0434-000 | WGS | ASD | 147855127 | A>G | Intron/NA | 1.285 | - |
| denovo-db | 2-0198-003 | WGS | ASD | 147891813 | C>G | Intron/NA | 1.412 | - |
| denovo-db | 1-0181-004 | WGS | ASD | 147966935 | T>C | Intron/NA | 3.955 | - |
| denovo-db | 2-1194-003 | WGS | ASD | 147976882 | A>G | Intron/NA | 1.19 | - |
| denovo-db | 5-0003-004 | WGS | ASD | 147993724 | AG>A | Intron/NA | -1 | - |
| denovo-db | 7-0059-003 | WGS | ASD | 147996378 | G>A | Intron/NA | 1.175 | - |
| denovo-db | 1-1004-003 | WGS | ASD | 148020651 | G>C | Intron/NA | 0.22 | - |
| denovo-db | 5-0003-003 | WGS | ASD | 148020840 | G>A | Intron/NA | 2.816 | - |
| denovo-db | 2-1510-003 | WGS | ASD | 148024345 | C>A | Intron/NA | 4.177 | - |
| denovo-db | 1-0294-003 | WGS | ASD | 148030697 | A>G | Intron/NA | 3.619 | - |
| denovo-db | 1-0022-003 | WGS | ASD | 148105328 | G>A | Intron/NA | 1.672 | - |
| denovo-db | 1-0022-004 | WGS | ASD | 148105328 | G>A | Intron/NA | 1.672 | - |
