# Supplementary material for: Comprehensive cross-disorder analyses of CNTNAP2 suggest it is unlikely to be a primary risk gene for psychiatric disorders
Source: PLoS Genet. 2018 Dec 26;14(12):e1007535. doi: 10.1371/journal.pgen.1007535 (PMC6324819; doi:10.1371/journal.pgen.1007535)
Supplement: S3 Table — (DOCX) [file pgen.1007535.s005.docx]

**S3 Table. Full list of Ultra-Rare Variants (URVs) found in publically available sequencing datasets.** This analysis included the Sweden-Schizophrenia population-based Case-Control cohort (SWE) (dbGAP accession: phs000473.v2.p2), Medical Genome Reference Bank (MGRB), BCM ASD case and control data set (BCM) and ASD cases from ARRA c1 data set (ARRA_c1; dbGAP accession: phs000298.v3.p2), a sequenced Spanish population [95, 96], and previous published sequence data in ASD [84]. All URVs are selected to be rare (MAF<0.0001 in Non-Finnish European population in gnomAD; <http://gnomad.broadinstitute.org/>) and predicted to be pathogenic both in SIFT and polyphen, with relative SIFT and polyphen scores provided. Genomic positions at chromosome 7 are indicated according to the GRCh37/hg19 assembly of the UCSC Genome Browser (genome.ucsc.edu).

| **Position** | **Ref/Alt** | **Data_set (Cases/Controls)** | **Type** | **gnomAD allele frequency** | **SIFT** | **Polyphen** | **Exon (1-24 exons)** | **Amino acid change** | **dbSNP ID** |
| --- | --- | --- | --- | --- | --- | --- | --- | --- | --- |
| 146471380 | C/T | SWE (1/0) | missense | 1.793e-05 | 0 | 0.998 | 2 | L/F | rs747896321 |
| 146536949 | G/A | SWE (1/0) | missense | 8.956e-06 | 0 | 0.964 | 3 | D/N | rs777035367 |
| 146536996 | G/A | SWE (1/0) | stop_gained,  splice_region | 8.972e-06 | - | - | 3 | W/* | rs761596436 |
| 146536997 | G/A | SWE (1/0) | splice_donor | 8.973e-06 | - | - | intr_3 | - | rs767408882 |
| 146805280 | T/C | SWE (1/0) | missense | 1.795e-05 | 0.02 | 0.979 | 5 | Y/H | rs764642738 |
| 146805395 | T/A | SWE (1/1) | missense | 1.793e-05 | 0 | 0.979 | 5 | I/N | rs749945304 |
| 146825798 | G/A | SWE (0/1) | missense | - | 0 | 0.98 | 7 | G/D | COSM5692203 |
| 146829415 | G/A | SWE (0/1) | missense | 3.588e-05 | 0.01 | 0.989 | 8 | G/R | rs775430816 |
| 146997232 | G/A | SWE (0/2) | splice_acceptor | 2.688e-05 | - | - | intr_8 | - | rs748180430 |
| 146997269 | G/T | SWE (1/0) | missense | 8.966e-06 | 0.05 | 0.561 | 9 | R/L | rs774717843 |
| 147092768 | G/C | SWE (1/0) | missense | 5.386e-05 | 0 | 0.95 | 10 | Q/H | rs535454043 |
| 147092817 | A/T | SWE (3/1) | missense | 3.593e-05 | 0 | 0.593 | 10 | R/W | rs781675293 |
| 147183037 | A/C | SWE (0/2) | missense | 3.581e-05 | 0 | 0.999 | 11 | N/H | rs764902194 |
| 147183107 | G/A | SWE (0/1) | missense | 0 | 0 | 0.897 | 11 | G/E | rs772138449 |
| 147259310 | G/A | SWE (1/0) | missense | 3.586e-05 | 0 | 1 | 12 | G/R | rs142980731 |
| 147336207 | T/A | SWE (1/0) | missense | 9.009e-06 | 0.02 | 0.477 | 13 | V/E | rs758867059 |
| 147336209 | T/C | SWE (2/0) | missense | 1.802e-05 | 0 | 0.558 | 13 | W/R | rs778170524 |
| 147600758 | C/T | SWE (0/2) | missense | 1.805e-05 | 0.03 | 0.786 | 14 | R/C | rs775506190 |
| 147600798 | C/T | SWE (0/2) | missense | 2.724e-05 | 0 | 0.843 | 14 | A/V | rs200214541 |
| 147675028 | G/A | SWE (1/0) | missense | 2.686e-05 | 0.01 | 0.927 | 15 | R/H | rs752756517 |
| 147675066 | C/T | SWE (1/3) | missense | 6.269e-05 | 0.02 | 0.886 | 15 | R/C | rs200089329 |
| 147815227 | G/A | SWE (1/0) | missense | 8.956e-06 | 0.02 | 0.923 | 16 | A/T | rs768132735 |
| 147815248 | T/C | SWE (1/0) | missense | 8.954e-06 | 0 | 0.961 | 16 | S/P | rs773802167 |
| 147815275 | G/A | SWE (0/1) | missense | 7.168e-05 | 0 | 0.737 | 16 | G/R | rs140239461 |
| 147844726 | C/T | SWE (3/1) | missense | 4.477e-05 | 0.02 | 0.892 | 17 | R/W | rs375318010 |
| 147844771 | C/T | SWE (1/0) | missense | 4.48e-05 | 0 | 0.959 | 17 | R/C | rs756798140 |
| 147869390 | G/C | SWE (0/1) | missense | 8.965e-06 | 0 | 1 | 18 | G/R | rs778815007 |
| 147869417 | G/C | SWE (0/1) | missense | 8.959e-06 | 0 | 0.98 | 18 | A/P | rs745938929 |
| 147869454 | G/A | SWE (0/1) | missense | 8.957e-06 | 0 | 0.878 | 18 | G/D | rs762274260 |
| 147869561 | T/G | SWE (0/1) | missense | - | 0 | 1 | 18 | C/G | rs779292118 |
| 147926773 | C/T | SWE (0/1) | stop_gained | 8.962e-06 | - | - | 20 | R/* | rs771533907 |
| 148080921 | T/TGAC  CCTCTCCCCC | SWE (0/1) | Indels_frameshift | 4.066e-6 |  |  |  | p.M1224Nfs*27 | rs770489662 |
| 148106478 | TACAG  CCA/T | SWE (4/1) | Indels | - |  |  |  |  | rs750631856 |
| 148106557 | A/T | SWE (2/0) | missense | 2.686e-05 | 0.01 | 0.953 | 23 | I/F | rs758146566 |
| 148112515 | T/C | SWE (0/1) | missense | 8.979e-06 | 0.02 | 0.982 | 24 | I/T | rs748244169 |
| 146805237 | A/T | c1_ARRA (0/5) | splice_acceptor | 0 | - | - | intr_4 | - | rs760761522 |
| 147092725 | C/G | c1_ARRA (0/1) | stop_gained | - | - | - | 10 | S/* | COSM1699532 |
| 147092844 | A/T | c1_ARRA (0/1) | missense | 8.99E-06 | 0 | 0.865 | 10 | S/C | rs140699088 |
| 147183111 | C/G | c1_ARRA (0/1) | stop_gained | - | - | - | 11 | Y/* | rs766920511 |
| 147259310 | G/A | c1_ARRA (0/1) | missense | 3.59E-05 | 0 | 1 | 12 | G/R | rs142980731 |
| 147926748 | G/T | c1_ARRA (0/1) | missense | 1.79E-05 | 0 | 0.998 | 20 | Q/H | rs773491586 |
| 147964128 | G/C | c1_ARRA (0/1) | missense | 0 | 0 | 0.958 | 21 | D/H | rs781236853 |
| 148106478 | TACAGCCA/T | c1_ARRA (0/1) | Indels_frameshift | - |  |  |  |  | rs750631856 |
| 147336315 | T/G | BCM_cases_controls (1/0) | missense | - | 0 | 0.514 | 13 | I/R | - |
| 147675066 | C/T | BCM_cases_controls (0/1) | missense | 6.27E-05 | 0.02 | 0.886 | 15 | R/C | rs200089329 |
| 147675079 | A/G | BCM_cases_controls (0/1) | missense ,splice_region | - | 0 | 0.989 | intr_15 | D/G | rs770270957 |
| 147815275 | G/A | BCM_cases_controls (0/1) | missense | 7.17E-05 | 0 | 0.737 | 16 | G/R | rs140239461 |
| 147844595 | T/A | BCM_cases_controls (0/1) | missense | - | 0 | 0.99 | 17 | V/E | - |
| 148080758 | C/T | BCM_cases_controls (1/0) | stop_gained | - | - | - | 22 | Q/* | - |
| 148080958 | G/A | BCM_cases_controls (0/1) | stop_gained | - | - | - | 22 | W/* | - |
| 146741012 | A/G | MGRB (1/0) | missense | 0 | 0.01 | 1 | 4 | N/S | rs370517200 |
| 146741032 | G/A | MGRB (1/0) | missense | 0 | 0 | 0.955 | 4 | V/I | rs781338949 |
| 146741113 | G/C | MGRB (1/0) | missense | - | 0 | 0.963 | 4 | G/R | COSM3635649 |
| 146741145 | C/A | MGRB (1/0) | stop_gained,  splice_region | - | - | - | 4 | Y/* | - |
| 146741147 | G/A | MGRB (1/0) | splice_donor | - | - | - | Intr_4 | - | - |
| 146818098 | G/GGA | MGRB (1/0) | frameshift |  |  |  |  |  |  |
| 146818118 | G/A | MGRB (1/0) | missense | - | 0 | 0.998 | 6 | G/R | - |
| 146997258 | G/A | MGRB (1/0) | stop_gained | - | - | - | 9 | W/* | - |
| 147259287 | A/G | MGRB (1/0) | missense | - | 0 | 0.969 | 12 | Y/C | - |
| 147336338 | G/A | MGRB (1/0) | missense | 0 | 0.02 | 0.801 | 13 | E/K | rs368905425 |
| 147815275 | G/A | MGRB (1/0) | missense | 0 | 0 | 0.737 | 16 | G/R | rs140239461 |
| 147869373 | G/A | MGRB (1/0) | missense | 4.50E-05 | 0 | 1 | 18 | R/H | rs1048353998 |
| 147869547 | A/G | MGRB (1/0) | missense | - | 0 | 0.971 | 18 | Y/C | - |
| 147926753 | G/A | MGRB (1/0) | missense | 0 | 0.04 | 0.511 | 20 | R/Q | rs201827086 |
| 147926846 | G/A | MGRB (1/0) | missense | 0 | 0 | 0.997 | 20 | R/H | rs774709566 |
| 148080896 | G/A | MGRB (1/0) | missense | 0 | 0.02 | 0.787 | 22 | E/K | rs768389541 |
| 146471423 | C/G | Murdoch et al. (0/1) | missense | 0 | 0.01 | 0.756 | 2 | S/C | rs762223005 |
| 146536932 | A/G | Murdoch et al. (0/1) | missense | 0 | 0 | 1 | 3 | Y/C | rs779208613 |
| 146536973 | T/A | Murdoch et al. (1/0) | missense | - | 0 | 0.999 | 3 | Y/N | - |
| 146741012 | A/G | Murdoch et al. (0/1) | missense | 0.00006267 | 0.01 | 1 | 4 | N/S | rs370517200 |
| 146805341 | C/T | Murdoch et al. (2/0) | missense | 0.00004484 | 0 | 0.998 | 5 | T/M | rs771028883 |
| 146805364 | C/A | Murdoch et al. (1/0) | missense | 0.00005379 | 0.01 | 0.717 | 5 | L/M | rs372345438 |
| 146818163 | C/T | Murdoch et al. (1/0) | missense | 0.000008972 | 0.04 | 0.999 | 6 | R/C | rs794727802 |
| 146825793 | T/G | Murdoch et al. (1/0) | missense | 0.00001794 | 0.04 | 0.752 | 7 | F/L | rs750383045 |
| 146829398 | G/A | Murdoch et al. (1/0) | missense | 0 | 0 | 0.968 | 8 | S/N | rs371839994 |
| 146997307 | G/A | Murdoch et al. (1/0) | missense | - | 0.01 | 0.858 | 9 | D/N | COSM4673361 |
| 147092781 | G/C | Murdoch et al. (1/0) | missense | - | 0.02 | 0.996 | 10 | D/H | COSM6109219 |
| 147336315 | T/G | Murdoch et al. (1/0) | missense | - | 0 | 0.514 | 13 | I/R | - |
| 147675066 | C/T | Murdoch et al. (0/1) | missense | 0.00006269 | 0.02 | 0.886 | 15 | R/C | rs200089329 |
| 147675070 | G/A | Murdoch et al. (1/0) | missense | - | 0 | 0.996 | 15 | C/Y | - |
| 147675079 | A/G | Murdoch et al. (0/1) | missense ,splice_region | - | 0 | 0.989 | 15 | D/G | rs770270957 |
| 147815275 | G/A | Murdoch et al. (0/1) | missense | 0.00007168 | 0 | 0.737 | 16 | G/R | rs140239461 |
| 147844595 | T/A | Murdoch et al. (0/1) | missense | - | 0 | 0.99 | 17 | V/E | - |
| 147844726 | C/T | Murdoch et al. (0/1) | missense | 0.00004477 | 0.02 | 0.892 | 17 | R/W | rs375318010 |
| 147844771 | C/T | Murdoch et al. (1/0) | missense | 0.0000448 | 0 | 0.959 | 17 | R/C | rs756798140 |
| 147926846 | G/A | Murdoch et al. (0/1) | missense | 0.000008961 | 0 | 0.997 | 20 | R/H | rs774709566 |
| 147964128 | G/C | Murdoch et al. (0/2) | missense | 0 | 0 | 0.958 | 21 | D/H | rs781236853 |
| 147964213 | T/C | Murdoch et al. (0/1) | missense | - | 0 | 0.978 | 21 | V/A | - |
| 147674967 | G/T | Spanish_server (1/0) | missense | - | 0.01 | 1 | 15 | G/C | rs755268329 |
| 147844744 | C/T | Spanish_server (1/0) | missense | 5.37E-05 | 0 | 0.985 | 17 | R/C | rs141617212 |
| 147914602 | T/A | Spanish_server (1/0) | missense | - | 0 | 0.968 | 19 | L/H | rs772512862 |
| 147964128 | G/C | Spanish_server (1/0) | missense | 0 | 0 | 0.958 | 21 | D/H | rs781236853 |
| 146805364 | C/A | Puente et al. (1/0) | missense | 0.00005379 | 0.01 | 0.717 | 5 | L/M | rs372345438 |
| 146997268 | C/T | Puente et al. (1/0) | missense | 0.00002689 | 0.01 | 0.935 | 9 | R/C | rs769071764, |
| 147964128 | G/C | Puente et al. (1/0) | missense | 0 | 0 | 0.958 | 21 | D/H | rs781236853 |
