# Supplementary material for: Comprehensive cross-disorder analyses of CNTNAP2 suggest it is unlikely to be a primary risk gene for psychiatric disorders
Source: PLoS Genet. 2018 Dec 26;14(12):e1007535. doi: 10.1371/journal.pgen.1007535 (PMC6324819; doi:10.1371/journal.pgen.1007535)
Supplement: S4 Table — (DOCX) [file pgen.1007535.s006.docx]

**S4 Table. Primers used in the CNV validation and genotyping qPCR experiment for the *CNTNAP2* intronic deletion in each member of the multiplex bipolar pedigree.** Two probes were designed for the deletion and two reference probes were used for normalization. Results from both CNTNAP2 probes were consistent with each other, and interpretation of copy number was identical when normalised by either reference probe.

| **Gene** | **Primer** | **Sequence 5'-3'** |
| --- | --- | --- |
| *CNTNAP_1* | F | ATACTATCGTCGTCATGCCAGA |
|  | R | TAAGCTGACTAGTGACACCCTG |
| *CNTNAP_2* | F | TTTGGAAGACTTGACATGGCAG |
|  | R | ACACAAAGTCAACAAAGCCAGT |
| *FOXP2* | F | TGCTAGAGGAGTGGGACAAGTA |
|  | R | GAAGCAGGACTCTAAGTGCAGA |
| *RNF20* | F | GAGACAGCCGAATCACGAGT |
|  | R | CCCAAGGTGAGTCTTCCTGA |

Abbreviations: F: Forward; R: Reverse
